# Interplay between Copper, Phosphatidylserine, and α-Synuclein Suggests a Link between Copper Homeostasis and Synaptic Vesicle Cycling

**DOI:** 10.1021/acschemneuro.4c00280

**Published:** 2024-07-16

**Authors:** Xiangyu Teng, Ewelina Stefaniak, Keith R. Willison, Liming Ying

**Affiliations:** † Department of Chemistry, Imperial College London, Molecular Sciences Research Hub, 82 Wood Lane, London W12 0BZ, U.K.; ‡ National Heart and Lung Institute, Imperial College London, Molecular Sciences Research Hub, 82 Wood Lane, London W12 0BZ, U.K.

**Keywords:** synaptic vesicle, α-synuclein, phosphatidylserine, copper homeostasis, kinetics

## Abstract

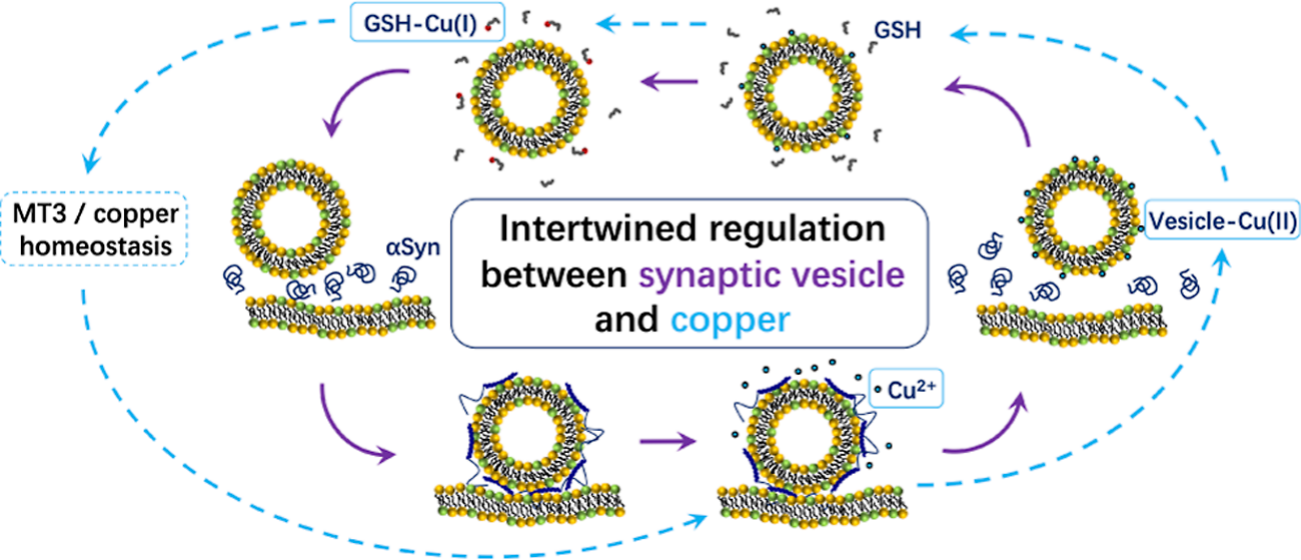

Copper homeostasis is critical to the functioning of the brain, and its breakdown is linked with many brain diseases. Copper is also known to interact with the negatively charged lipid, phosphatidylserine (PS), as well as α-synuclein, an aggregation-prone protein enriched in the synapse, which plays a role in synaptic vesicle docking and fusion. However, the interplay between copper, PS lipid, and α-synuclein is not known. Herein, we report a detailed and predominantly kinetic study of the interactions among these three components pertinent to copper homeostasis and neurotransmission. We found that synaptic vesicle-mimicking small unilamellar vesicles (SUVs) can sequester any excess free Cu^2+^ within milliseconds, and bound Cu^2+^ on SUVs can be reduced to Cu^+^ by GSH at a nearly constant rate under physiological conditions. Moreover, we revealed that SUV-bound Cu^2+^ does not affect the binding between wild-type α-synuclein and SUVs but affect that between N-terminal acetylated α-synuclein and SUVs. In contrast, Cu^2+^ can effectively displace both types of α-synuclein from the vesicles. Our results suggest that synaptic vesicles may mediate copper transfer in the brain, while copper could participate in synaptic vesicle docking to the plasma membrane via its regulation of the interaction between α-synuclein and synaptic vesicle.

## Introduction

Copper is a trace element found in all body tissues and is required for many cellular functions. Copper is present at high levels in the central nervous system (CNS) and is of great importance for the normal maturity and function of the brain. Copper is involved in a variety of physiological processes, such as cellular respiration and neurotransmitter biosynthesis and most notably serves as a cofactor of many enzymes responsible for CNS development.^1,2^ Intracellular brain copper concentration is 2 to 3 orders of magnitude higher than its extracellular counterpart.^3^ Notably, nearly all brain copper is associated with protein, and its concentration and distribution among different subcellular compartments are tightly controlled.^3,4^ Furthermore, copper has been known to bind strongly to negatively charged lipid, phosphatidylserine (PS), which is a major lipid species of synaptic vesicle.^5,6^ This could lead to a hypothesis that the synaptic vesicle may take part in brain copper homeostasis.

Extensive studies suggest that the precise maintenance of the levels of copper is crucial, and the loss of it is one of the prominent features across many neurodegenerative disorders including Parkinson’s disease (PD) and Alzheimer’s disease (AD).^7,8^ In PD, the links between copper dysregulation and other cellular processes are known to play a role in dopamine oxidation, oxidative stress, and α-synuclein (αSyn) aggregation.^9^

αSyn is a ∼ 14 kDa intrinsically disordered protein mainly located in presynaptic terminals with an abundance equivalent to ∼50 μM concentration.^10^ Its aggregation is associated with PD and other neurodegenerative diseases known as synucleinopathies.^11,12^ In vivo, αSyn exists in an equilibrium between a membrane-bound state and a soluble unstructured form. The N-terminal region of the αSyn sequence has been shown to adopt the conformation of an amphipathic α helix promoting membrane binding.^13^ The physiological function of αSyn is still under debate, but significant evidence exists about the potential role of αSyn in the regulation of the synaptic vesicle release and synaptic plasticity.^14−16^

The precise mechanism that induces the abnormal aggregation of αSyn is not yet fully established. Extensive research has indicated that αSyn mutation, posttranslational modification, lipid membrane, and metal ion interactions can all accelerate αSyn aggregation.^17−20^ Among metal cations present in the brain, copper is the most effective in accelerating aggregation; hence, preserving or restoring copper homeostasis is of great importance and among the not-well-explored therapeutic routes for PD.^21^ Moreover, the excess reactive oxygen species (ROS) generated via the redox cycling of Cu^2+^/Cu^+^ in the presence of αSyn oligomers is considered as a potential contributor to the molecular mechanism of promoting the onset of PD by copper dyshomeostasis.^22^

We have recently investigated the effect of N-terminal acetylation and a familial PD mutation on the kinetics of copper binding to αSyn by the stopped flow technique.^23^ Given that few studies have explored the role of copper in synaptic transmission and plasticity^24^ and that both copper and αSyn bind to synaptic vesicles, we have tackled the binding of copper to the synaptic membrane and its impact on αSyn association with membrane by performing fast kinetics measurements. Such key information has been neglected for a long time, and our approach can determine the key reaction rate constants for the chemical events likely occurring in the synaptic cleft during neurotransmission.

Considering the significance of copper imbalance and its involvement in neurodegenerative diseases, we have also investigated amyloid-β (Aβ) peptide in the same setting. Aβ, thought to be central to the pathogenesis of AD, undergoes aggregation and produces ROS in the presence of copper ions.^25,26^ During synaptic transmission, both copper (in concentration of up to 250 μM)^27,28^ and the Aβ peptide are released to the glutamatergic synapses, where Aβ aggregation is proposed to take place.^29,30^ Kinetics of high-affinity copper binding by Aβ^31,32^ and other extracellular proteins, such as human serum albumin (HSA), may influence the availability of this cation for cell uptake, causing abnormal copper redistribution, thus leaving the cells deficient of copper.^33^ An individual cell, under physiological conditions, contains on average less than one labile copper ion,^34^ while under pathological conditions, shuttling of copper to its specific cellular targets by intracellular chaperons is disrupted. After entering the cells, copper is sequestered by GSH. This prevents ROS generation by free copper, thereby lowering copper toxicity within the cell.^35,36^ However, the Cu^+^-GSH pair is still redox-active and can constantly react with molecular oxygen to produce superoxide.^37,38^ Nevertheless, the susceptibility of cells to copper toxicity correlates strongly with their cellular GSH content. Despite or perhaps because of the complexity of this balance, a large array of factors can limit cellular capability to maintain copper balance and redox homeostasis.^39^ It should be emphasized that the participation of GSH in copper regulation and neural transmission^40^ as well as its protective role for neural cells against excitotoxicity are not fully addressed. Furthermore, the rationale for the synaptic vesicles to store copper pool is still unclear.^41^

Herein, we report an extensive investigation into the interactions between copper, PS lipid, and αSyn on synaptic-like lipid membrane by stopped flow and other spectroscopic methods. We have determined the kinetics of Cu^2+^ binding to 50 nm synaptic-like small unilamellar vesicles (SUVs) and revealed the influence of GSH, HSA, as well as Aβ peptide and αSyn. In parallel, we have evaluated the kinetics of αSyn binding to SUVs and how Cu^2+^ binding to SUVs affects such kinetics. Moreover, we obtained the reduction kinetics of the SUV-Cu^2+^ conjugate in the presence of GSH. Our results suggest that, on the one hand, synaptic vesicles may mediate copper transfer in the brain, while on the other hand, copper could play a role in the regulation of synaptic vesicle docking to the plasma membrane.

## Results

### Cu^2+^ Binding to Synaptic-Like Vesicles

**Figure 1:**
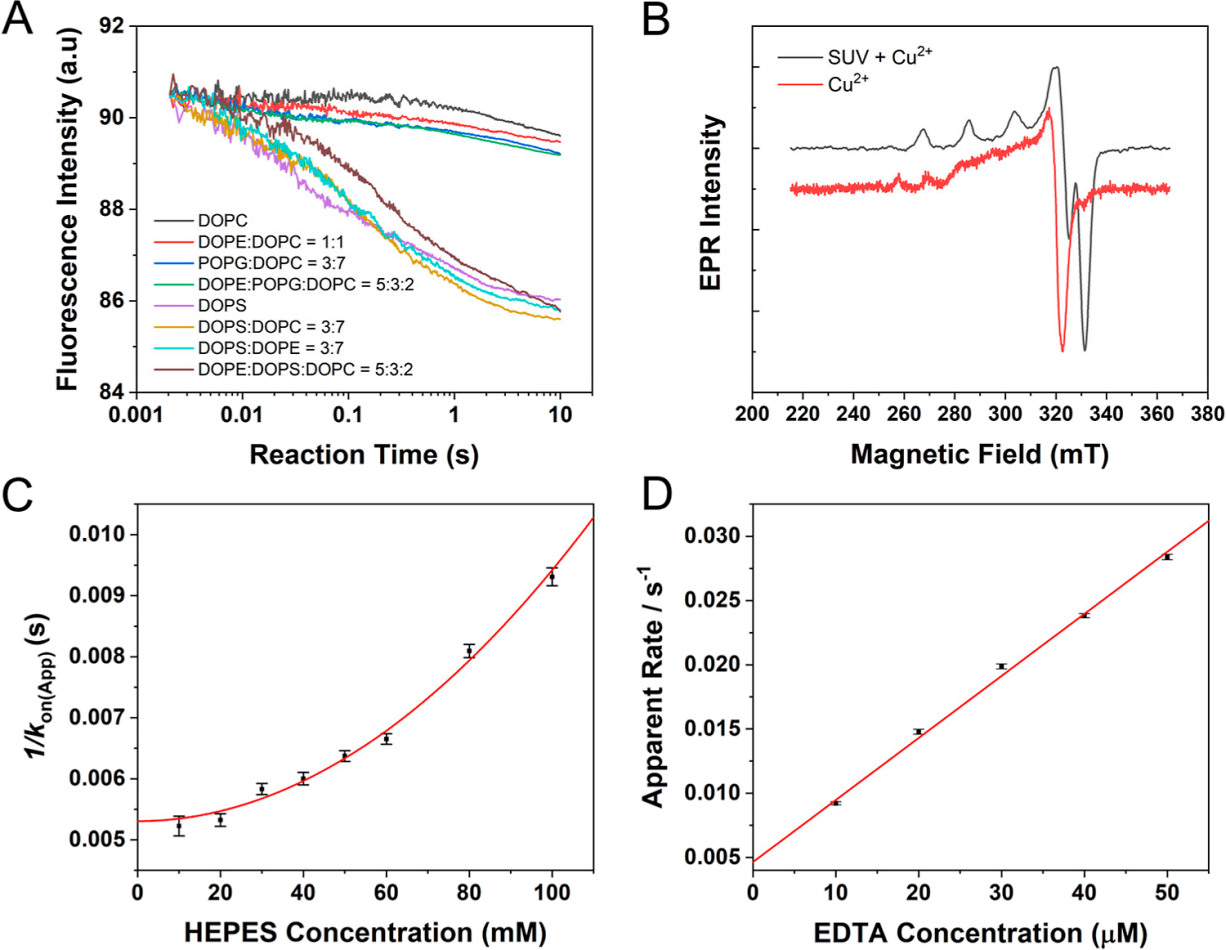
Interactions between Cu^2+^ and synaptic-like SUVs. (A) Stopped flow reaction time profiles of 500 nM Cu^2+^ with NBD-labeled SUVs (100 μM total lipid) with various lipid compositions at 298 K. (B) X-band EPR spectra of 50 μM Cu^2+^ in the presence and absence of synaptic-like SUVs (15 mM total lipid). (C) Kinetics of Cu^2+^ binding to synaptic-like SUVs under different HEPES concentrations. The HEPES-independent binding rate constant was derived from the *Y*-intercept of an empirical zero-centered parabola fitting (red curve). (D) Kinetics of the reaction of EDTA with SUV-Cu^2+^. The linear fit (red line) was used to derive the second-order reaction rate constant (slope) and the spontaneous dissociate rate of the conjugate (*Y*-intercept). All experiments were performed in 50 mM HEPES buffer (pH 7.5) containing 100 mM NaCl, except for those for the measurement of HEPES dependence of the binding reaction where the HEPES concentration was varied from 10 to 100 mM, while the NaCl concentration stayed at 100 mM.

To investigate the interactions between synaptic-like SUV and Cu^2+^, 50 nm nitrobenzoxadiazole (NBD)-labeled fluorescent SUVs were prepared. The SUVs were composed of DOPE, DOPS, and DOPC in a lipid content ratio of 5:3:2 w/w to mimic synaptic vesicles. NBD is an environmentally sensitive fluorescence probe widely used in lipid membrane studies.^42,43^ Emission spectra of NBD-labeled SUVs (1 mM total lipid) with and without adding 100 μM CuCl_2_ clearly show fluorescence quenching upon the addition of Cu^2+^ (Figure S1A), indicating that Cu^2+^ can bind to synaptic-like SUVs. Fluorescence correlation spectroscopy (FCS) measurements were also performed to confirm that the quenching was not due to vesicle disruption (Figure S1B). As synaptic-like SUVs contain DOPE, DOPS, and DOPC, it was necessary to identify which lipid constituent in the SUVs interacted with Cu^2+^. Labeled SUV samples containing various lipid compositions (100 μM total lipid) were mixed with 500 nM CuCl_2_, and the binding reaction traces were recorded as a function of time by stopped flow. As shown in Figure 1A, only SUVs containing DOPS displayed significant fluorescence quenching, suggesting that DOPS is the Cu^2+^-reactive lipid constituent in synaptic-like SUVs.

Next, an X-band EPR measurement was carried out to determine the Cu^2+^ binding mode to synaptic-like SUVs. The spectra are shown in Figure 1B. A clear difference can be seen between the spectra of Cu^2+^ in the presence and absence of synaptic-like SUVs, indicating Cu^2+^ binding to SUVs. The *g*_∥_ factor and hyperfine coupling constant (*A*_∥_) of SUV-bound Cu^2+^ obtained by spectral simulation were 2.262 and 203 G, respectively. These parameters are in good agreement with a 2N2O binding mode according to the Peisach–Blumberg plot^44^ and is also consistent with a previous Cu^2+^/lipid binding study using the POPC/DOPS lipid system, which identified DOPS as a strong Cu^2+^ binder with affinity in the picomolar regime.^45^

The binding kinetics of Cu^2+^ to synaptic-like SUVs was subsequently studied by performing stopped flow measurements, and the Cu^2+^ association rate constant (*k*_on_) was first determined. The NBD-labeled SUVs (100 μM total lipid containing 30 μM DOPS) were mixed with 1 μM CuCl_2_ under various HEPES concentrations to derive HEPES-independent *k*_on_, as described previously.^46^ The raw traces (Figure S2A) were fitted (fitting is described in Methods) to obtain the apparent association rates (*k*_on(App)_). Buffer-independent *k*_on_ was then derived to be 6.3(3) × 10^6^ M^–1^ s^–1^ from the intercept of an empirical zero-centered parabola fitting of *k*_on(App)_^–1^ against different HEPES concentrations (Figure 1C). This rate constant indicates that any labile Cu^2+^ ions would be sequestered by synaptic vesicles within a millisecond. Next, to obtain the spontaneous Cu^2+^ dissociation rate constant (*k*_off_) of the SUV-Cu^2+^ conjugate, labeled SUVs (100 μM total lipid) were premixed with 1 μM CuCl_2_ to form the SUV-Cu^2+^ conjugate. Then the solution was treated with various concentrations of ethylenediamine tetraacetic acid (EDTA). The raw traces are shown in Figure S2B. Linear fitting of the apparent rates gave the value of *k*_off_ to be 4.6(5) × 10^–3^ s^–1^ from the intercept, and the second-order rate constant for the reaction between SUV-Cu^2+^ and EDTA to extract Cu^2+^ to be 313(5) M^–1^ s^–1^ from the slope (Figure 1D). The apparent equilibrium dissociation constant (*K*_d_) of SUV-Cu^2+^ was calculated by ratioing the *k*_off_ by *k*_on_, giving the value of 0.7(1) nM.

### Reduction Kinetics of the SUV-Cu^2+^ Conjugate

The high Cu^2+^ binding affinity of synaptic-like SUVs may suggest that the synaptic vesicle could play an important role in copper transportation and regulation. To further address this question, we investigated the reduction of the SUV-Cu^2+^ conjugate following a previously reported kinetic method.^23^ Both ascorbate and glutathione (GSH) were selected as reductants. Labeled SUVs (100 μM total lipid) were premixed with 1 μM CuCl_2_ to form the SUV-Cu^2+^ conjugate; then the solution was treated with various concentrations of sodium ascorbate. Interestingly, the traces do not show any fluorescence recovery (Figure S3A), indicating that the SUV-Cu^2+^ conjugate is reduction-inert for ascorbate.

**Figure 2:**
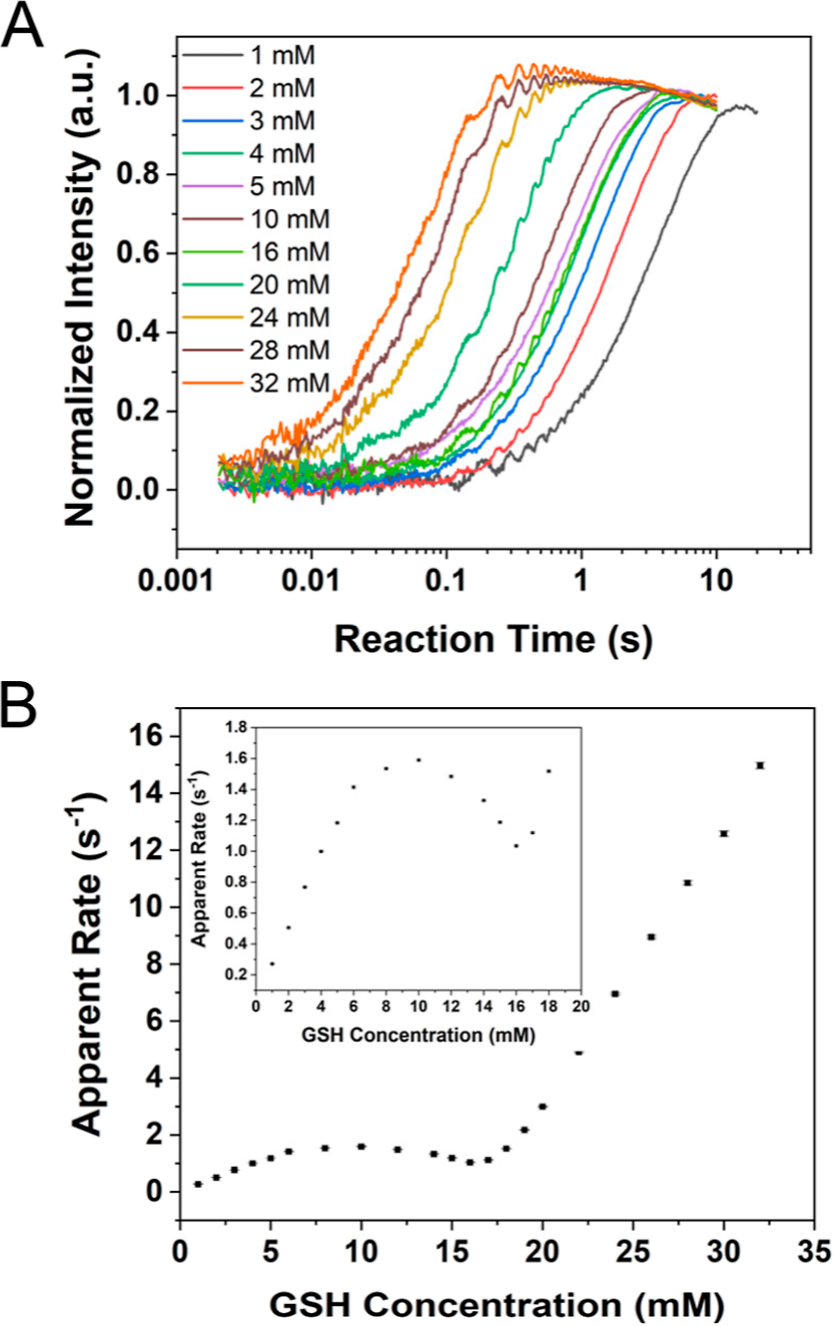
Reduction kinetics of the SUV-Cu^2+^ conjugate by GSH. (A) Reaction traces of the SUV-Cu^2+^ conjugate (100 μM total lipid, 1 μM Cu^2+^) with GSH. The experiments were performed in 50 mM HEPES buffer (pH 7.5) containing 100 mM NaCl at 298 K. (B) GSH concentration dependence of apparent reduction rates. The inset is an expanded view of the plot with GSH concentration below 20 mM for clarity.

Next, the reduction of the same SUV-Cu^2+^ conjugate by GSH was conducted. As shown in Figures 2A and S3B, GSH can efficiently reduce the SUV-Cu^2+^ conjugate, indicating a possibility that the reductive metabolism of Cu^2+^ on synaptic vesicle could involve the glutathione/glutathione disulfide (GSH/GSSG) pair, which is closely linked to copper homeostasis.^47^ The derived apparent reduction rates from single exponential fitting of raw traces are shown in Figure 2B. Intriguingly, these rates exhibit a peculiar characteristic upon increasing GSH concentration. The reduction rate increases slowly and linearly as the GSH concentration rises until it reaches 5 mM. The rate increase is then slowed down until it reaches a local maximum and then decreases. When the GSH concentration is over 16 mM, the reduction rate rises fast and seems to be “out of control”. The same trend was also observed on GSH reduction of the αSyn-Cu^2+^ complex (Figure S4). We note that the upper limit of the physiological GSH concentration reported so far is approximately 15 mM.^48−51^ Therefore, the reduction rate is only weakly dependent on the physiological GSH level.

### Cu^2+^ Binding Competition between Synaptic-like SUVs and Proteins

There are multiple Cu^2+^ binding proteins in neurons. The study of Cu^2+^ binding competition between synaptic-like SUVs and Cu^2+^ binding proteins is consequently important to understand the process and mechanism of copper regulation in the brain. Herein, three common neuronal Cu^2+^ binding proteins, Aβ, HSA, and αSyn, were selected for investigation.

**Figure 3:**
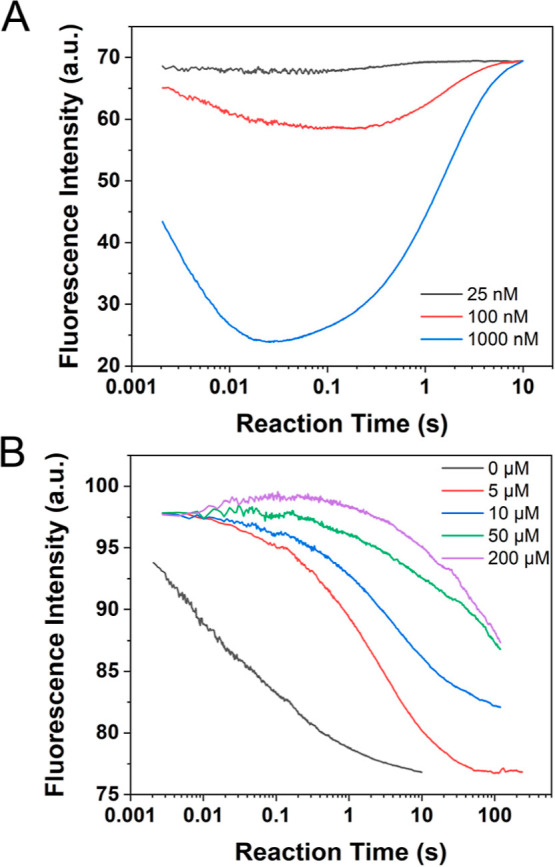
Competition of Cu^2+^ binding between synaptic-like SUVs and Aβ_40_ as well as HSA. (A) Competition between 25 nM Aβ_40_ and SUVs (100 μM total lipid) for Cu^2+^ binding under various Cu^2+^ concentrations. (B) Competition between HSA and SUVs for Cu^2+^ binding (100 μM total lipid, 5 μM Cu^2+^) under various concentrations of HSA. Experiments were performed in 50 mM HEPES buffer (pH 7.5) containing 100 mM NaCl at 298 K.

We first measured the competition of Cu^2+^ binding between synaptic-like SUV and Aβ using stopped flow. 25 nM HiLyte Fluor 488-labeled Aβ_40_ was premixed with synaptic-like SUVs (100 μM total lipid); then the mixture was treated with different concentrations of CuCl_2_. As shown in Figure 3A, Aβ_40_ is initially bound to Cu^2+^. However, Cu^2+^ is then extracted by SUVs at longer timescales as the fluorescence of Aβ_40_ is recovered. Similar competition traces are presented on other Aβ species (Figure S5). As the amplitude of fluorescence quenching of Aβ in the presence of SUVs is smaller than that of Aβ in free solution, the factual competition process can be described as the following: Aβ and SUVs initially bind to Cu^2+^ independently; afterward, SUVs start to extract Cu^2+^ from the Aβ-Cu^2+^ complex. In case of the competition with Aβ_4–16_, Cu^2+^ extraction seems to occur on the Aβ_4–16_-Cu^2+^ intermediates rather than the final Aβ_4–16_-Cu^2+^ complex as the final Aβ_4–16_-Cu^2+^ complex has been shown to be formed after ∼2 s of the initial Cu^2+^ binding,^23^ but here, Cu^2+^ extraction by SUVs starts at ∼0.01 s after initial binding. We also performed an FCS measurement to confirm that Aβ has negligible interaction with synaptic-like SUVs when the lipid concentration is below 1 mM (Figure S6).

As a major Cu^2+^ carrier in the blood and a common protein in the brain, HSA may also affect the Cu^2+^ binding to synaptic vesicles. Therefore, a Cu^2+^ competition experiment between HSA and SUV was conducted. A control measurement was first performed by FCS to determine the binding of synaptic-like SUVs with fluorescent-labeled HSA, which only shows weak HSA binding on SUVs. A small increase of diffusion time was observed compared to that of SUV, rendering the fraction of HSA bound to SUVs to be ∼4% (Figure S7). In the competition experiment, the labeled SUVs (100 μM total lipid) were premixed with various concentrations of HSA; then the mixture was mixed with 5 μM CuCl_2_. Figure 3B shows that the presence of HSA can slow down Cu^2+^ binding to SUVs but cannot stop it. This suggests that initially HSA can compete against SUV in the binding of Cu^2+^. However, at longer timescales, Cu^2+^ will be transferred to SUVs, as indicated by the decrease of fluorescence from the labeled SUVs. Considering that the Cu^2+^ binding affinity of HSA (reported as 0.1 pM^52^) is much higher than that of SUVs (0.7(1) nM), Cu^2+^ transfer is expected to occur on the nascent HSA-Cu^2+^ intermediates after the initial Cu^2+^ binding rather than via the final HSA-Cu^2+^ complex, a scenario similar to the competition process of Cu^2+^ binding between Aβ_4–16_ and SUVs, as described above. If the experiment was performed under physiological HSA concentration (5 μM) in cerebrospinal fluid (CSF), such competition can also be observed (Figure S8A).

In a separate experiment where the preformed SUV-Cu^2+^ conjugate was reacted with HSA, no fluorescence recovery was observed (Figure S8B), indicating that HSA cannot extract Cu^2+^ from SUV-Cu^2+^ on the timescale of the stopped flow measurement. This result may suggest that Cu^2+^ coordination in the HSA-Cu^2+^ intermediate state is much weaker than that in the SUV-Cu^2+^ conjugate, thus making HSA unable to extract Cu^2+^ from the latter. Both observations are consistent with the presence of a more reactive and less stable HSA-Cu^2+^ intermediate after initial Cu^2+^ binding to HSA but in disagreement with the order of the thermodynamic stability of the final complexes.

**Figure 4:**
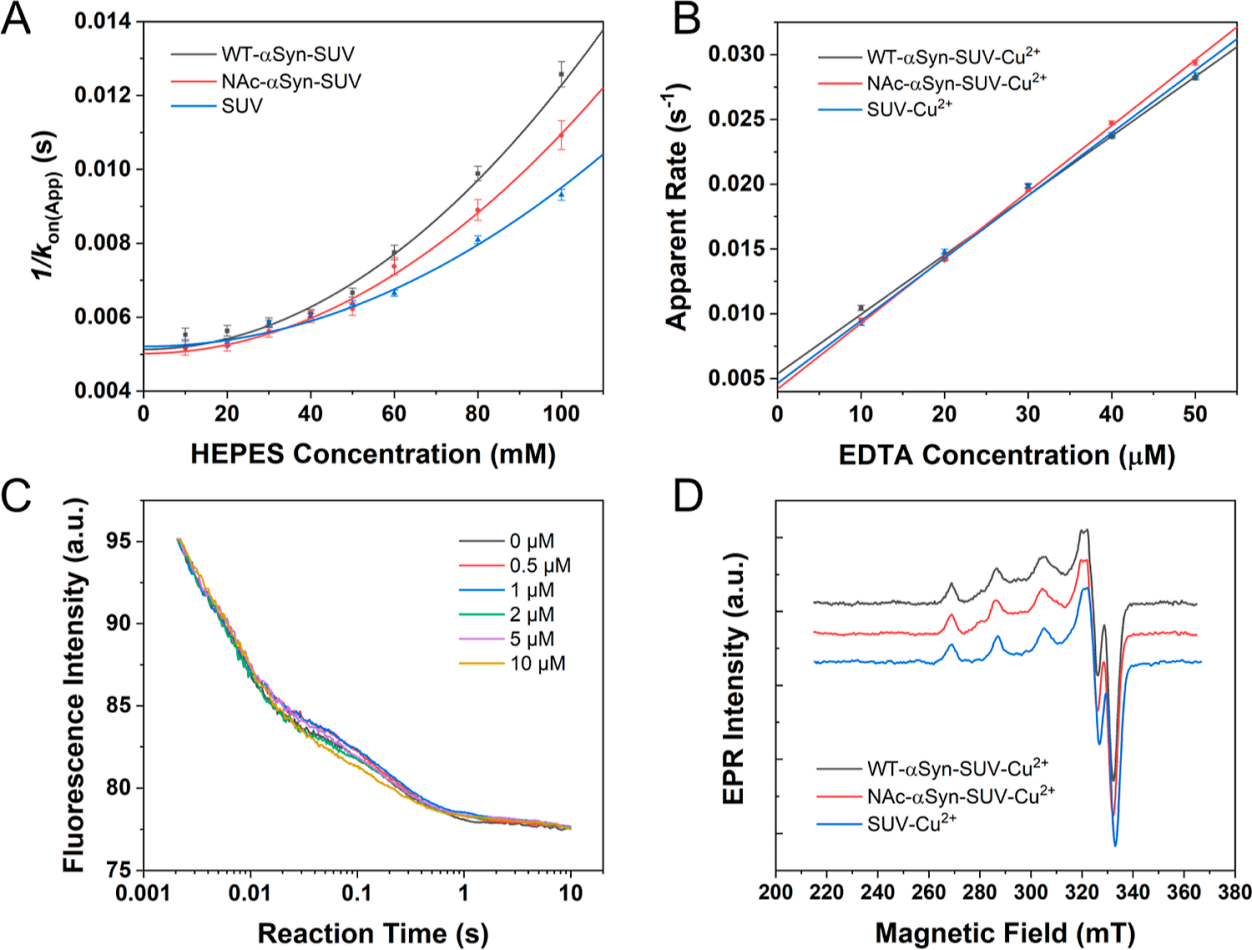
Negligible impact of αSyn on Cu^2+^ binding to αSyn-SUV conjugates. (A) HEPES concentration dependence of the kinetics of Cu^2+^ binding to αSyn-SUV (both WT and NAc forms) conjugates and SUVs. (B) Kinetics of the reaction of EDTA with αSyn-SUV-Cu^2+^ (both WT and NAc forms) and SUV-Cu^2+^. (C) Cu^2+^ (1 μM) binding to WT-αSyn-SUV under great excess of WT-αSyn (various αSyn concentrations). (D) X-band EPR spectra of Cu^2+^ (50 μM) bound on αSyn-SUV (both WT and NAc forms, 50 μM αSyn with 15 mM total lipid) and SUVs. All figures present evidence that αSyn does not participate in the Cu^2+^ binding to αSyn-SUV conjugates. All experiments were performed in 50 mM HEPES buffer (pH 7.5) containing 100 mM NaCl, except for those for the measurement of HEPES dependence of the binding reaction where the HEPES concentration was varied from 10 to 100 mM while the NaCl concentration stayed at 100 mM.

We then moved to assess the capability of Cu^2+^ binding between synaptic-like SUVs and αSyn. Unlike Aβ and HSA, αSyn can readily bind to synaptic-like SUVs (Figure S9). Thus, Cu^2+^ competition between them would proceed on the SUVs. In this experiment, both wild-type αSyn (WT-αSyn) and N-terminal acetylated αSyn (NAc-αSyn) were studied by stopped flow. 25 nM labeled αSyn was premixed with synaptic-like SUVs (100 μM total lipid); then 1 μM CuCl_2_ was blended in the mixture under various concentrations of HEPES buffer. The raw reaction traces (Figure S10A,B) are virtually identical. Strikingly, the traces are also similar to those for Cu^2+^ binding to SUVs (Figure S2A). Indeed, the derived *k*_on_ values of Cu^2+^ binding to WT-αSyn-SUV and NAc-αSyn-SUV, 6.4(7) × 10^6^ and 6.6(5) × 10^6^ M^–1^ s^–1^, respectively (Figure 4A), are statistically identical and cannot be differentiated from the value of 6.3(3) × 10^6^ M^–1^ s^–1^ for *k*_on_ of Cu^2+^ binding to SUVs.

Such similar reaction traces and close *k*_on_ values between Cu^2+^ binding to αSyn-SUV conjugates and SUVs may suggest that the SUV-bound αSyn is not the actual species binding to Cu^2+^. Instead, the labeled αSyn may simply serve as a fluorescent probe indicating how Cu^2+^ binds to SUVs. To further validate this assumption, the spontaneous Cu^2+^ dissociation rate constants (*k*_off_) of αSyn-SUV-Cu^2+^ conjugates were then determined. αSyn-SUV-Cu^2+^ conjugates were prepared by mixing 25 nM labeled αSyn, synaptic-like SUVs (100 μM total lipid), and 1 μM Cu^2+^. The mixture was then treated with various concentrations of EDTA. The reaction traces are shown in Figure S10C,D. Plotting the derived apparent rate against the EDTA concentration, *k*_off_ values were determined from the intercepts of fitted data, which are 5.3(5) × 10^–3^ and 4.2(1) × 10^–3^ s^–1^ for WT-αSyn-SUV-Cu^2+^ and NAc-αSyn-SUV-Cu^2+^, respectively (Figure 4B). These values are quite close to the value of 4.6(5) × 10^–3^ s^–1^ for *k*_off_ of the SUV-Cu^2+^ conjugate but significantly different from the *k*_off_ of the αSyn-Cu^2+^ complexes [0.017(4) s^–1^ for WT-αSyn-Cu^2+^ and 0.10(1) s^–1^ for NAc-αSyn-Cu^2+^].^23^ Therefore, we conclude that it is SUV rather than SUV-bound αSyn that participated in Cu^2+^ binding.

In the measurements above, the αSyn concentration was in the nanomolar regime, and the binding between αSyn and synaptic-like SUVs was not saturated. To study whether excessive αSyn can affect Cu^2+^ binding to SUVs, various micromolar concentrations of αSyn (both WT and NAc forms) were added to the labeled SUV (100 μM total lipid, ∼ 4 nM SUVs) sample. At such concentrations of αSyn, all vesicles should be fully bound with αSyn, and there will be excess αSyn in the solution.^53^ Cu^2+^ binding traces are shown in Figure 4C for WT-αSyn/SUVs and Figure S11 for NAc-αSyn/SUVs. The traces remain identical even at high micromolar concentrations of αSyn, indicating that excessive αSyn cannot prevent or compete Cu^2+^ binding to SUVs. This suggests that the N-terminal Cu^2+^ binding site of SUV-bound αSyn is buried within the membrane and thus no longer available for Cu^2+^ binding, which is consistent with a recently proposed membrane binding configuration for the first 14 N-terminal residues of αSyn.^54^ Furthermore, as SUVs possess a faster Cu^2+^ binding rate and stronger Cu^2+^ binding affinity than αSyn,^23^ free αSyn has no chance to compete the binding of Cu^2+^ against SUVs. This result also indicates that even in the presence of excessive αSyn, some DOPS lipids on SUVs are unshielded by the protein and hence still able to bind Cu^2+^.

To further strengthen the conclusion that αSyn does not participate in Cu^2+^ binding when it coexists with synaptic-like SUVs, an X-band EPR measurement was conducted. Figure 4D shows the comparison of the EPR spectra for SUV-Cu^2+^, WT-αSyn-SUV-Cu^2+^, and NAc-αSyn-SUV-Cu^2+^ conjugates. All spectra are the same, suggesting that the chemical environments of Cu^2+^ in these three conjugates are identical, which unequivocally confirms that Cu^2+^ can only bind to DOPS lipids even in the presence of αSyn on SUVs.

### Cu^2+^ Can Detach αSyn from αSyn-SUV Conjugates

**Figure 5:**
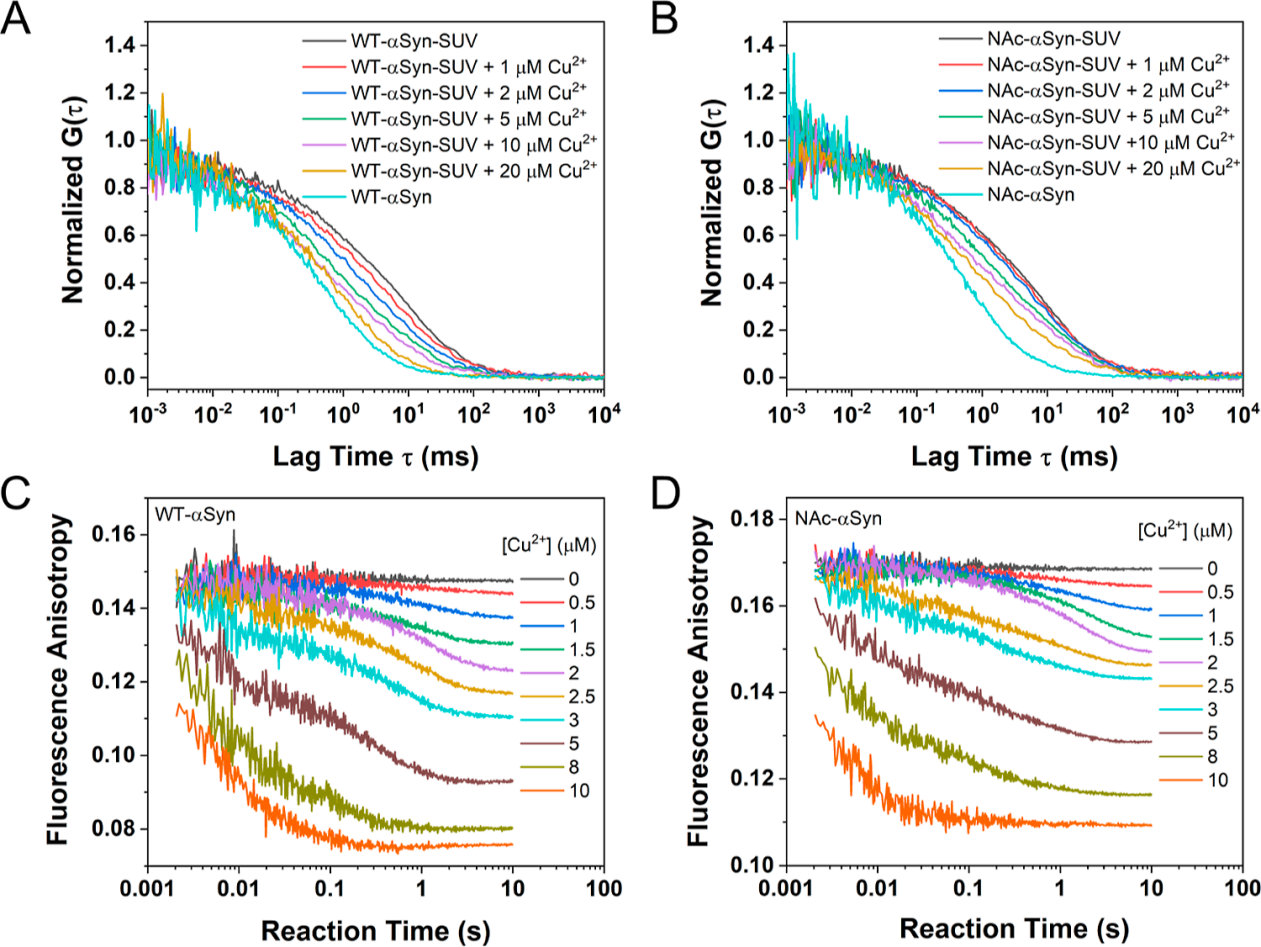
Detachment of αSyn from αSyn-SUV by Cu^2+^. FCS curves of SUV-bound WT-αSyn (A) and NAc-αSyn (B) under various concentrations of Cu^2+^, and fluorescence anisotropy kinetic curves of SUV-bound WT-αSyn (C) and NAc-αSyn (D) under various concentrations of Cu^2+^ show that Cu^2+^ can detach αSyn from αSyn-SUV conjugates. The experiments were performed in 50 mM HEPES buffer (pH 7.5) containing100 mM NaCl at 298 K.

After establishing that SUV-bound αSyn does not affect Cu^2+^ binding to SUV, we next sought to address whether Cu^2+^ can affect the binding of SUV-bound αSyn. We first performed FCS measurements on 10 nM labeled αSyn (both WT and NAc forms) premixed with synaptic-like SUV (100 μM total lipid) and then treated with various concentrations of CuCl_2_. The normalized FCS curves are shown in Figure 5A,B. FCS characterization of the mixture indicates that αSyn detachment from the αSyn-SUV conjugate is Cu^2+^ concentration dependent, and the introduction of Cu^2+^ promotes αSyn dissociation from the αSyn-SUV conjugate. The same conclusion can also be made from the time profiles of fluorescence anisotropy. In these stopped flow measurements, 25 nM labeled αSyn (both WT and NAc forms) was mixed with 100 μM lipid and incubated for 10 min; then the mixture was treated with various concentrations of CuCl_2_. Fluorescence anisotropy time traces (Figure 5C,D) exhibit decreasing trend in the presence of Cu^2+^, with the rate of such a decrease dependent on the Cu^2+^ concentration. Furthermore, Cu^2+^ titration monitored by steady-state fluorescence anisotropy supports the same conclusion (Figure S12C,D).

To determine whether other divalent metal ions influence the stability of the αSyn-SUV conjugate likewise, Ca^2+^ and Zn^2+^ were investigated in the same way. High micromolar concentrations of metal ions were employed to mimic their physiological concentrations. As shown in Figures S13–S16, no changes were observed to the FCS curves and anisotropy values of SUV-bound αSyn at a wide range of Ca^2+^ and Zn^2+^ concentrations tested, suggesting that Ca^2+^ and Zn^2+^ exert hardly any influence on αSyn binding to synaptic-like SUVs.

### αSyn Binding to SUVs Loaded with Cu^2+^

**Figure 6:**
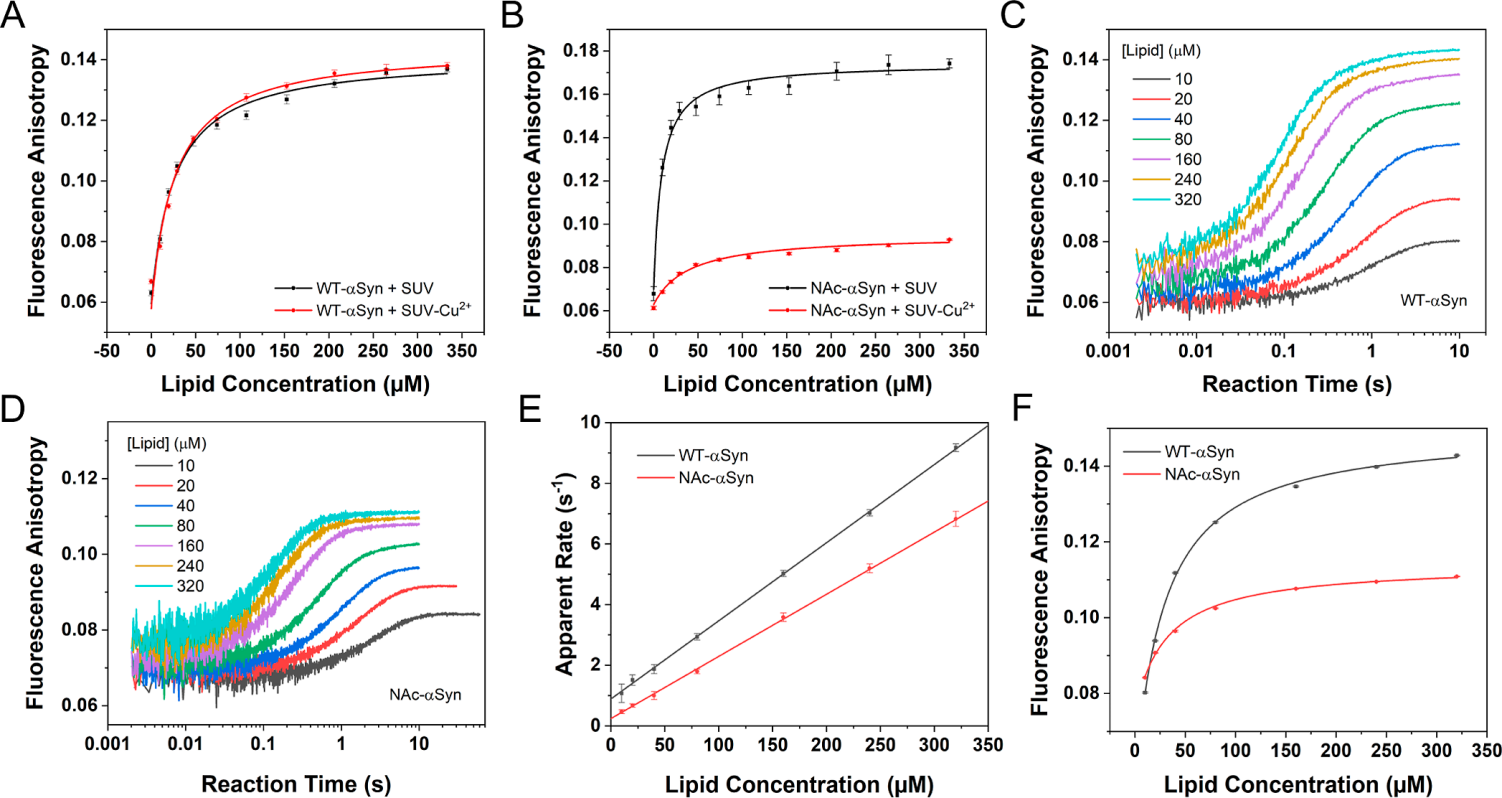
αSyn binding to SUV-Cu^2+^ (Cu^2+^: SUV lipid = 1:100). (A) Fluorescence anisotropy titration of WT-αSyn binding to SUV-Cu^2+^. (B) Fluorescence anisotropy titrations of NAc-αSyn binding to SUV-Cu^2+^. (C) Fluorescence anisotropy kinetics of WT-αSyn binding to SUV-Cu^2+^. (D) Fluorescence anisotropy kinetics of NAc-αSyn binding to SUV-Cu^2+^. (E) Apparent binding rates of αSyn to SUV-Cu^2+^ derived from fluorescence anisotropy kinetic curves. (F) Final anisotropy from the fits of fluorescence anisotropy kinetic curves as a function of SUV concentration (shown in the concentrations of lipid). The plots were fitted to derive the binding affinity values of αSyn to SUV-Cu^2+^, giving similar results to the values determined by anisotropy titration experiments. All experiments were performed in 50 mM HEPES buffer (pH 7.5) containing 100 mM NaCl at 298 K.

The work described above indicates that Cu^2+^ can enhance the dissociation of αSyn from synaptic-like SUVs. We next addressed the question whether Cu^2+^ can influence αSyn binding to SUVs if SUV membranes are associated with Cu^2+^. An FCS measurement was performed to rule out the potential effect of Cu^2+^ binding on the size of SUVs (Figure S1B). Fluorescence anisotropy titration experiments were then conducted to evaluate the binding affinity of αSyn with SUVs associated with Cu^2+^. In these experiments, CuCl_2_ was blended in SUV solution to a ratio of 1:100 (Cu^2+^/SUV lipid) to form SUV-Cu^2+^ conjugates. Then 100 nM labeled αSyn was titrated against various concentrations of Cu^2+^-mixed lipid, and meanwhile, fluorescence anisotropy was monitored. Panels A and B of Figure 6 show the titration results for WT-αSyn and NAc-αSyn, respectively, in the presence and absence of Cu^2+^. An obvious difference in the anisotropy can be observed for the binding of NAc-αSyn to SUVs loaded with and without Cu^2+^. The dissociation constant (*K*_d_) for NAc-αSyn binding to the SUV-Cu^2+^ conjugate is 40(8) μM, five times larger than that for the binding to SUV [8(2) μM], suggesting that the SUV-bound Cu^2+^ weakens the binding of NAc-αSyn to SUVs. Surprisingly, the binding of WT-αSyn to SUVs is less affected by Cu^2+^, as indicated by the identical dissociation constant determined [*K*_d_ for WT-αSyn-SUV-Cu^2+^ and WT-αSyn-SUV are 26(6) μM and 26(5) μM, respectively]. This may imply that while bound Cu^2+^ on SUVs does not seem to affect the membrane binding conformation of WT-αSyn, it does so for NAc-αSyn, perhaps reducing its membrane penetration.

We also studied the reaction kinetics of αSyn binding to SUV-Cu^2+^ by monitoring the time profile of fluorescence anisotropy. The Cu^2+^-loaded lipid sample was prepared as described above, and 50 nM labeled αSyn (both WT and NAc forms) was treated with various concentrations of this sample. The anisotropy time traces were recorded as shown in Figure 6C,D. By fitting these traces with multiexponentials, the apparent binding rates of αSyn binding to the SUV-Cu^2+^ conjugate were obtained, which are plotted against the concentration of Cu^2+^-mixed lipid in Figure 6E. The binding rate constants (*k*_on_), as determined from the slopes of fits, are 3.0(1) × 10^4^ and 2.21(5) × 10^4^ M^–1^ s^–1^ for WT-αSyn and NAc-αSyn, respectively. Compared with *k*_on_ for αSyn binding to SUV [3.3(1) × 10^4^ M^–1^ s^–1^ for WT-αSyn and 2.19(7) × 10^4^ M^–1^ s^–1^ for NAc-αSyn] (data shown as Figure S17), the values for the binding to SUV-Cu^2+^ are similar, indicating that SUV-bound Cu^2+^ does not impact the binding rate of αSyn to SUVs. In addition, by fitting the plateau values of the kinetic traces (Figure 6F), the *K*_d_ for αSyn binding to SUV-Cu^2+^ can also be determined, which are 27(4) μM and 34(6) μM for WT-αSyn and NAc-αSyn, respectively. These values are in good agreement with those determined by fluorescence anisotropy described earlier.

## Discussion

PS is known to bind to Cu^2+^ with high affinity by forming a tight 2:1 PS-Cu^2+^ complex.^6,45,55^ Here, we studied the binding kinetics between Cu^2+^ and a synaptic vesicle mimicking SUV system that contains DOPS, DOPC, and DOPE. We found that the association and dissociation rate constants are on the 10^6^ M^–1^ s^–1^ and 10^–3^ s^–1^ order, respectively. The synaptic-like SUV binds Cu^2+^ in a 2N2O binding mode with an apparent *K*_d_ around 0.7 nM. In comparison, the apparent *K*_d_ for Cu^2+^ binding to PS-containing supported lipid bilayer (SLB) was determined to be 6.4 pM for SLB containing 20% PS.^6^ The discrepancy in the binding affinity may arise from the difference in the lipid environment and experimental condition. Since the surface potential of a negatively charged lipid bilayer can enhance the effective Cu^2+^ concentration in the electrical double layer, steady-state measurement observed an enhancement of binding affinity by several orders of magnitude.^6^ We expected that in the stopped flow measurement where the fast-binding reaction would prevent the buildup of the Cu^2+^ concentration near the vesicle surface, such an effect would not be as pronounced as that under equilibrium. Indeed, we observed a higher apparent equilibrium dissociation constant even though the vesicles we used contain a higher percentage of PS than that of the literature report.

Strikingly, PS can compete with both Aβ_4–16_ and HSA for Cu^2+^ binding on the second timescale because of the reaction between PS and the nascent intermediates where Cu^2+^ has not yet formed the 4N coordination with the protein/peptide.^32^ On the longer timescale where the competition reaches equilibrium, we expect that Cu^2+^ ions would eventually be trapped by PS where they form tight 2:1 stoichiometry complexes with an effective binding affinity equivalent to or higher than that of Aβ_4–16_ or HSA. Therefore, kinetics of the competition suggest that if Aβ and HSA coexist with SUVs, they can initially compete with the binding of Cu^2+^ ions that are transiently available during neurotransmission against SUVs. However, the bound Cu^2+^ on them will be transferred to SUVs, possibly via a weakly copper-coordinated intermediate state. Such intermediate states have already been discovered for Aβ and other peptides.^32,46,56^

One key property of the vesicle-bound Cu^2+^ lies in that its redox activity is significantly reduced in comparison to Cu^2+^ coordinated with Aβ peptide. This observation is in line with the redox activity of Cu^2+^ coordinated with αSyn.^23^ Since PS-coordinated Cu^2+^ cannot be reduced by ascorbate (redox potential of DHA/ASc couple vs SHE at pH 7 is 0.09 V) but rather by GSH (redox potential of GSSG/GSH couple vs SHE at pH 7 is −0.23 V),^57^ the redox potential of PS-Cu^2+^ would rest between 0.09 and −0.23 V at neutral pH. Consequently, ROS production from the redox cycling of Cu^2+^/Cu^+^ would be prohibited since the reduced product Cu^+^ will be effectively chelated by GSH, which can deliver it to copper transporter metallothionein.^58^ In addition to the favorable redox potential, significant affinity of GSH with Cu^+^ may account in part for the efficient reduction observed in the presence of excess GSH. It was reported that GSH and Cu^+^ form a highly stable oligomeric complex Cu_4_(GSH)_6_.^59,60^ Our observation manifests the well-recognized role of glutathione in regulating intracellular copper homeostasis.^40^ For instance, redox reaction of GSH regulates the incorporation of copper and maturation of CuA and CuB sites in COX, which are important steps in the mitochondrial respiratory chain.^40^ Based on our results, both PS lipids on synaptic vesicles and GSH likely participate in such regulation in the brain. We are aware that a recent study has indicated that the presence of membrane-like environments induces the formation of a 2:1 αSyn-Cu^+^ complex where Cu^+^ is bound to the Met1 and Met5 residues of the two helical peptide chains from the two protein molecules, where Cu^+^ is stabilized and is redox silenced.^61^ However, in the presence of a physiological concentration of GSH/GSSG, copper is more likely associated with GSH after reduction.

One remarkable characteristic of Cu^2+^ associated with synaptic-like vesicle is that its reduction rate remains weakly dependent on GSH across its physiological concentration range. This is important as local intracellular GSH concentrations could be quite anisotropic. This experimental evidence seems to suggest that synaptic vesicle binding might play a role in tightly regulating this pivotal redox reaction in copper homeostasis, even though further investigation would be needed to elucidate the mechanism responsible for such reaction kinetics and its significance.

**Figure 7:**
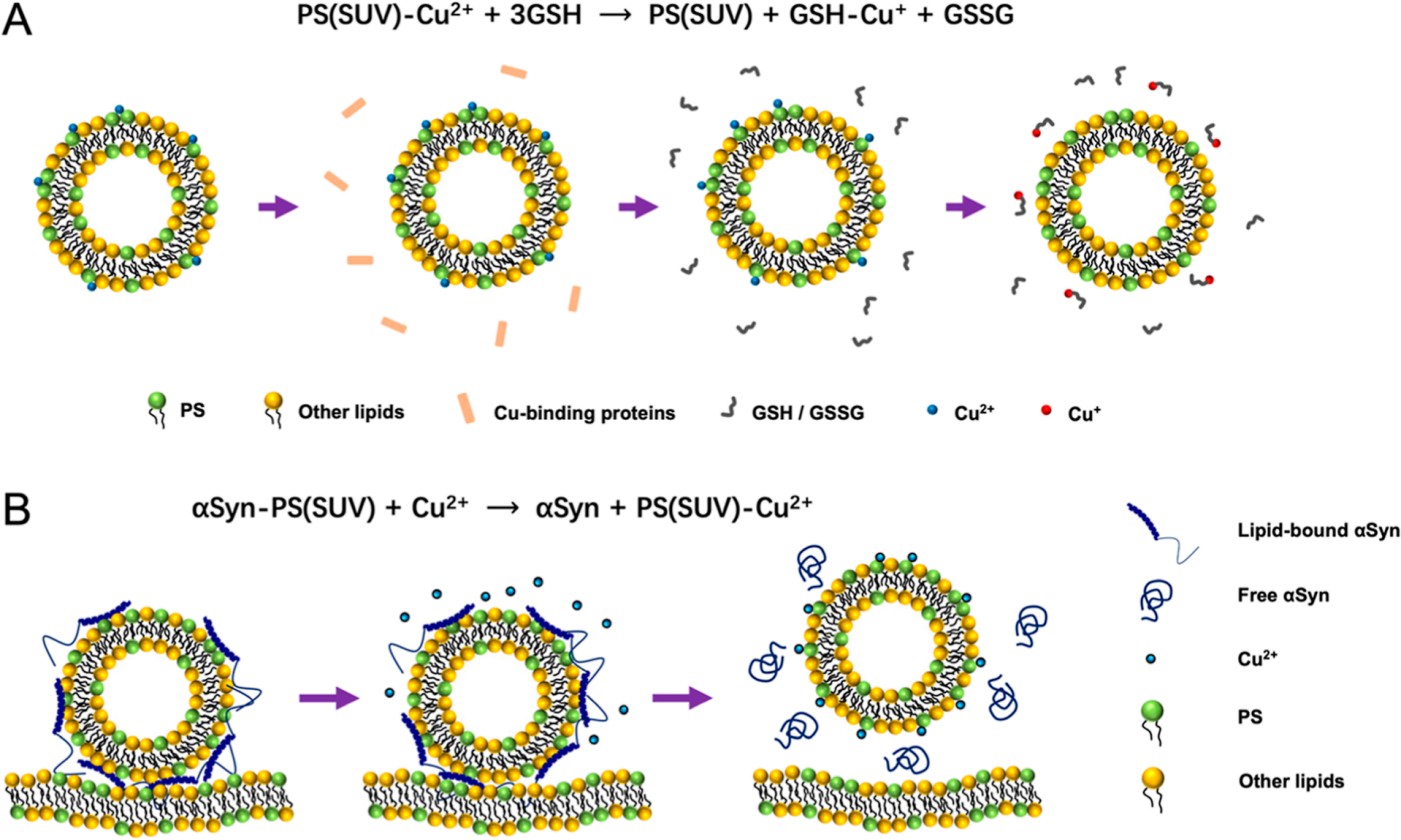
Schematics illustrating (A) synaptic vesicle-mediated copper transportation and (B) potential regulatory role of copper in αSyn-mediated synaptic vesicle trafficking and docking.

Taken together, we propose that synaptic vesicles may mediate copper transfer in the synapse, as illustrated in Figure 7A. Under physiological conditions, synaptic vesicles can sequester any excess Cu^2+^ within a millisecond and potentially hold the ions for up to 200 s, thereby protecting them from being taken by other Cu-binding proteins or peptides. This ensures that copper can be transported securely to the cellular locations where it is needed. The bound Cu^2+^ is reduced to Cu^+^ by GSH at a nearly constant rate under physiological conditions and then chelated by GSH. Next, Cu^+^ is transferred from GSH to copper transporters, such as MT3.

We have also investigated how the interactions between αSyn, synaptic-like SUV, and Cu^2+^ are influenced by one of the three that stands alone while the other two are bound together. Membrane-bound αSyn (both wild-type and N-terminal acetylated) does not seem to participate in binding to Cu^2+^, which can be explained by the membrane insertion of the first 14 residues of the N-terminus of αSyn,^54^ thus blocking the Cu^2+^ binding site of the protein. Furthermore, WT-αSyn binds to the SUV-Cu^2+^ conjugate and SUV alone with similar affinities, whereas NAc-αSyn exhibits significantly weaker binding to the SUV-Cu^2+^ conjugate than that to SUV. The association rate constants of αSyn with the SUV-Cu^2+^ conjugate are almost the same as those with SUV, implying that bound Cu^2+^ on SUVs does not significantly affect the membrane binding of αSyn kinetically. In contrast, when αSyn-SUV is exposed to free Cu^2+^, Cu^2+^ does affect the binding between αSyn and SUVs, as indicated by the detachment of αSyn from the SUVs as determined by FCS and fluorescence anisotropy measurements. Since the binding of Cu^2+^ ions to PS lipids triggers the deprotonation of their NH_3_^+^ groups, the net charge of the lipids remain unchanged.^6^ This may explain similar binding rate constants between αSyn and SUVs with or without Cu^2+^ as electrostatic interaction plays a major role in αSyn membrane binding.^62^ The weakening of αSyn binding to the membrane by Cu^2+^ is probably caused by the change in membrane rigidity after Cu^2+^ binding. Given that the N-terminal anchor (first 14 residues) of αSyn drives αSyn membrane interaction and insertion,^54^ it is likely that binding of Cu^2+^ ions to PS headgroups stiffens the membrane, making the membrane penetration of the anchor more difficult, especially for NAc-αSyn where the penetration of the anchor is deeper than that for WT-αSyn.^54^ Consequently, the dissociation of αSyn from SUV-Cu^2+^ is faster, thereby reducing the binding affinity (ratio between the dissociation rate constant and the association rate constant) since the association rate constant is not affected by Cu^2+^.

What could we learn from these new observations? We propose that Cu^2+^ might play a role in the regulation of synaptic vesicle docking to the plasma membrane. As we know, αSyn is a key participant in synaptic vesicle fusion and release.^63,64^ Moreover, a high concentration of αSyn inhibits the release of synaptic vesicles.^64^ A more recent study indicates that the docking of synaptic vesicles on the presynaptic membrane induced by αSyn is modulated by lipid composition and that changes in the lipid composition associated with neurodegenerative diseases alter the binding modes of αSyn.^16^ Since Cu^2+^ can weaken the binding between αSyn and SUVs, especially between the physiologically more relevant NAc-αSyn and SUVs, leading to the dissociation of αSyn from the membrane, we could postulate a scenario as illustrated in Figure 7B. In the presynaptic terminal, the motion of a synaptic vesicle is normally restricted by abundant NAc-αSyn, the physiological form of the protein. Once the neurotransmission is triggered by an action potential, copper is released to the synaptic cleft from synaptic vesicles.^65^ Transient copper concentration could also be high around the synaptic vesicle waiting for its release, enabling copper to coordinate with membrane constituents of synaptic vesicles, such as PS,^45^ hence playing a role in modulating synaptic membrane structure and function. Consequently, some αSyn molecules may leave from this synaptic vesicle, making it more mobile and ready to release. These processes could occur on the timescale of a millisecond, closely matching the rate of synaptic transmission.^66^ This hypothesis links the dynamic interactions among the synaptic vesicle, αSyn, and copper together to narrate the story of presynaptic vesicular trafficking and highlights the potential regulatory role of Cu^2+^ in synapse from a kinetically sound perspective.

We recognize that in the hypothesis described above, PS lipids would need to be available in the outer leaflet of the synaptic vesicle to interact with transiently released Cu^2+^. It is well documented that cell membranes have asymmetric lipid distribution where PS lipids are predominantly located at the cytoplasmic side of the membrane (inner leaflet).^67^ However, there is no consensus in the literature regarding the exact asymmetric distribution of PS lipids in synaptic vesicles, with the measured ratio of PS lipids between outer and inner leaflet ranging from 0:100 to 69:43.^68−70^ The disparity between these studies is likely caused by the different methodologies used. Nevertheless, such lipid asymmetry would be broken in neurotransmission. During vesicle fusion, phospholipid scramblase-1 is expected to randomize the lipid distribution;^71^ while during vesicle recycling, two PS lipids, one from the inner leaflet and the other from the outer leaflet, are required to cooperatively bind to both cytoplasmic and intravesicular lysine–arginine clusters in synaptogyrin for the formation of small vesicles.^72^ Therefore, regardless of the exact nature of asymmetry in PS lipid distribution in synaptic vesicle before neurotransmission, Cu^2+^ ions would be able to interact with PS transiently during the process of synaptic vesicle release and recycling. Further studies are desirable to provide more insights into this less recognized role of copper in neurotransmission in addition to its known role of inhibiting the NMDA receptor.^73^

We have also tested two well-studied metal ion players in neurotransmission, Zn^2+^ and Ca^2+^, yet no evidence has been found supporting that they would also weaken the interaction between αSyn and synaptic-like SUVs. Nevertheless, Ca^2+^ can bind to the C-terminus of αSyn and enhance its synaptic vesicle binding capacity.^74^ As such, both Cu^2+^ and Ca^2+^ might jointly influence the timing of the release of synaptic vesicles.

Finally, the observation of the displacement of αSyn by Cu^2+^ on the synaptic-like membrane points to a viable approach to inhibit the lipid membrane-mediated aggregation of αSyn, and therefore, a new direction for therapeutic development against synucleinopathies, such as PD. Indeed, squalamine, a natural cationic steroid, was reported to be able to inhibit αSyn aggregation and suppress its toxicity.^10^ More recently, a small-molecule compound, UCB0599, currently in phase 2 clinical trial for PD, was shown to change the ensemble of membrane-bound structures of αSyn.^75^ In this regard, we envisage that small-cell-penetrating cationic peptides might be good candidates as drug leads against PD.

## Conclusions

In this study, we have demonstrated that DOPS-containing synaptic-like SUVs can efficiently and quickly bind to Cu^2+^ in a 2N2O binding mode (*K*_d_ = 0.7 nM, *k*_on_ = 6.3 × 10^6^ M^–1^ s^–1^). The formed SUV-Cu^2+^ conjugate is too redox-inert to be reduced by ascorbate. Nevertheless, it can be efficiently reduced under physiological concentrations of GSH. In addition, bound Cu^2+^ on Aβ or HSA will be eventually transferred to SUVs even though Aβ and HSA can initially compete the binding of Cu^2+^ against SUVs. These results lead us to propose that the synaptic vesicle itself could act as a major Cu^2+^ carrier and transporter in the brain where its binding to Cu^2+^ can guide copper to the correct homeostasis pathway.

We have also investigated how αSyn, synaptic-like SUV, and Cu^2+^ interact with each other, from the view of one species interacting with the conjugate formed by the other two species. We have shown that αSyn does not affect synaptic-like SUV to bind Cu^2+^, and SUV-bound Cu^2+^ does not influence the rate of αSyn binding to the lipid membrane. One key finding is that free Cu^2+^ can displace both WT-αSyn and NAc-αSyn from the synaptic-like membrane. Furthermore, bound Cu^2+^ on SUV can also significantly weaken the binding between physiologically predominant NAc-αSyn, leading to the dissociation of αSyn from the membrane. These observations prompt us to hypothesize that apart from the variation of lipid composition, Cu^2+^ may also be involved in the αSyn-mediated synaptic vesicle docking to the plasma membrane. Our study further suggests that small cell-penetrating cationic peptide targeting might be a viable therapeutic approach against PD.

## Methods

### Preparation of SUVs

Coagulation Reagent I [DOPE/DOPS/DOPC (5:3:2 w/w)] was purchased from Avanti Polar Lipids as lyophilized powder. The lipid was dissolved in chloroform and then aliquoted and dried under a nitrogen stream to form lipid films. The lipid films were left under nitrogen flow overnight to remove any remaining solvent and then stored at −20 °C. To prepare SUVs, the lipid films were first hydrated in buffer solution with agitation for 1 h. Then the obtained lipid suspension was extruded through a polycarbonate membrane with 50 nm pores (Whatman) 15 times using a Mini-Extruder (Avanti Polar Lipids). The vesicle size was confirmed by dynamic light scattering using Zetasizer Ultra (Malvern Panalytical). The fluorescent SUV sample was prepared by mixing NBD-DOPE [1,2-dioleoyl-*sn*-glycero-3-phosphoethanolamine-*N*-(7-nitro-2–1,3-benzoxadiazol-4-yl), headgroup labeled, Avanti Polar Lipids] with the DOPE:DOPS:DOPC (5:3:2 w/w) lipid mixture at a molar ratio of 1:1000.

### Expression and Labeling of αSyn

WT-αSyn and NAc-αSyn were expressed and labeled as previously described.^23^ Briefly, pT7–7 asyn WT plasmid (Addgene plasmid # 36046) alone and both the pT7–7 asyn WT plasmid and the pNatB (pACYCduet-naa20-naa25) plasmid (Addgene plasmid # 53613) were used to produce WT-αSyn and NAc-αSyn, respectively, using BL21(DE3) *Escherichia coli* (Thermo Fisher Scientific). A glycine to cysteine mutation at position 7 was introduced to the protein using a Phusion Site-Directed Mutagenesis Kit (Thermo Fisher Scientific) for site-specific fluorescent labeling by Alexa Fluor 488C_5_ maleimide (Thermo Fisher Scientific). The final labeled αSyn concentration was determined from the absorbance at 495 nm with an extinction coefficient of 72 000 M^–1^cm^–1^, and the labeling efficiency was determined to be 95%. The labeled αSyn samples were stored at −80 °C.

### Stopped Flow Kinetics

All kinetic measurements were performed on a KinetAsyst SF-610 × 2 stopped flow spectrophotometer (HI-TECH Scientific). Samples were excited by a fiber-coupled MCLS1–473–20 diode laser at 473 nm (Thorlabs). Fluorescence emission was filtered using a 515 nm long pass filter (Comar) before being detected by a photon multiplier tube. Data were recorded using a logarithmic timescale sampling scheme, and a minimum of nine repeats were averaged. Data points below 2 ms were excluded in analysis to avoid the influence of the instrument dead time. The same instrument was employed to record the reaction time profiles in fluorescence anisotropy using the optional fluorescence anisotropy detection unit. Reaction curves obtained from the kinetic measurements under pseudo first-order conditions were fitted by either a single or double exponential function.^23^ In the latter case, the mean rate values (*k*_mean_) were taken as the apparent reaction rates. To determine HEPES buffer independent Cu^2+^ binding rate constant *k*_on_, the inverses of the apparent reaction rates at different HEPES concentrations were empirically fitted with a parabola centered at zero, and the intercept at the *Y*-axis was 1/*k*_on_. The second-order binding rates between αSyn and lipid membranes were derived from the slope of linear plot of the apparent binding rates vs lipid concentrations.

### Fluorescence Correlation Spectroscopy

FCS measurements were performed on a custom-built confocal microscope based on an inverted optical microscope (Eclipse TE2000-U, Nikon) equipped with a high numerical aperture objective (CFI Apochromat TIRF 60×, NA 1.49, Nikon). A tunable argon ion laser (35LAP321–230, Melles Griot) was used as the excitation light source. The fluorescence from the confocal volume passing through the confocal pinhole was split by a 50:50 beam splitter and detected by two detectors (SPCM-AQR-14 single photon counting module, PerkinElmer). Pseudoauto correlation function was generated by a digital hardware correlator (Flex02–01D/C, Correlator.com). Correlation curves were fitted by a 2D diffusion model of one or two species, and the diffusion times were obtained.

### Binding of αSyn to SUV Measured by Fluorescence Anisotropy Titration

Fluorescence anisotropy titration was performed on a spectrofluorometer (FluoroMax-4, Horiba) to determine the binding isotherm of αSyn to SUV. Anisotropy data as a function of lipid concentration were fitted by the Hill–Langmuir equation to determine the binding affinity assuming noncooperative binding.

### EPR Spectroscopy

CW EPR spectra of Cu^2+^ conjugates were detected with a Bruker EMX 300 EPR spectrometer equipped with a high sensitivity X-band (ca. 9.4 GHz) resonator and a liquid helium cryostat. Field corrections were applied by measuring relevant EPR standards (Bruker Strong Pitch and DPPH). For accuracy, the tube size and tube position in the cavity were kept constant. Sample solution was transferred into an EPR tube (4 mm o.d.) via micropipettes, and then the tube was placed into a 5 mm o.d. tube, which was perched with argon gas and sealed by a silicone plug. Then the sample was frozen in liquid nitrogen and transferred into a cryostat to cool down to 20 K. CW EPR spectra were recorded at a microwave power of ∼7 mW, modulation frequency of 100 kHz, and modulation amplitude of 10 G. Simulation of the EPR spectra was performed with the EasySpin/MATLAB toolbox, which employs the exact diagonalization of the spin Hamiltonian matrix.^76^

## Supporting Information Available

Data from control experiments as well as from kinetic experiments (PDF)
