# Supplementary material for: Interplay between Copper, Phosphatidylserine, and α-Synuclein Suggests a Link between Copper Homeostasis and Synaptic Vesicle Cycling
Source: ACS Chem Neurosci. 2024 Jul 16;15(15):2884–96. doi: 10.1021/acschemneuro.4c00280 (PMC11311125; doi:10.1021/acschemneuro.4c00280)
Supplement: Supplementary file 1 — cn4c00280_si_001.pdf [file cn4c00280_si_001.pdf]

Supporting Information

Interplay between Copper, Phosphatidylserine and α-Synuclein Suggests a Link between Copper Homeostasis and Synaptic Vesicle Cycling

Xiangyu Teng, Ewelina Stefaniak, Keith R. Willison, Liming Ying

AUTHOR INFORMATION

Corresponding author

Liming Ying — National Heart and Lung Institute, Imperial College London, London SW3 6LY, United Kingdom. ORCID: 0000-0001-9752-6292.

Email: l.ying@imperial.ac.uk.

Authors

Xiangyu Teng — Department of Chemistry, Imperial College London, London SW7 2AZ, United Kingdom.

Ewelina Stefaniak — National Heart and Lung Institute, Imperial College London, London SW3 6LY, United Kingdom.

Keith R. Willison — Department of Chemistry, Imperial College London, London SW7 2AZ, United Kingdom.

Supplementary Figures (Figure S1 to Figure S17)

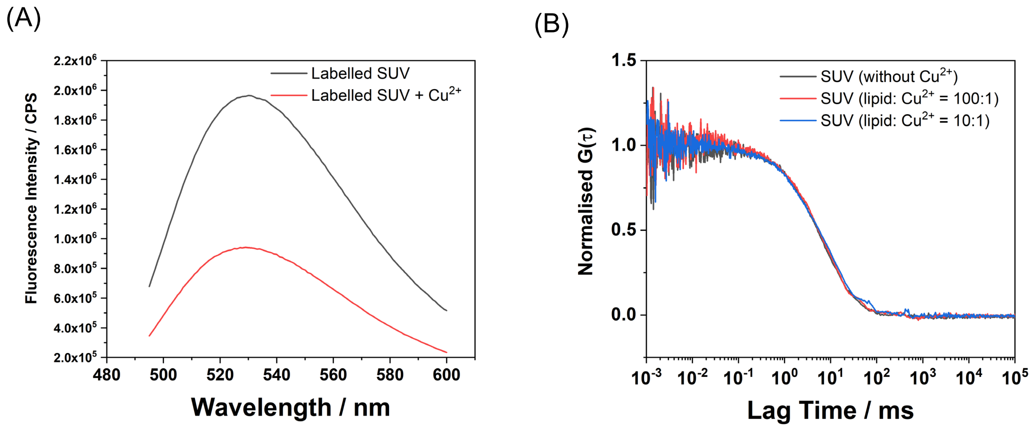

Figure S1. (A) Emission spectra of NBD labelled SUVs in the presence and absence of Cu^{2+}. (B) FCS curves showing that Cu^{2+} binding does not affect the size of SUVs. The experiments were performed in 50 mM HEPES and 100 mM NaCl buffer solution at 298 K (pH 7.5).

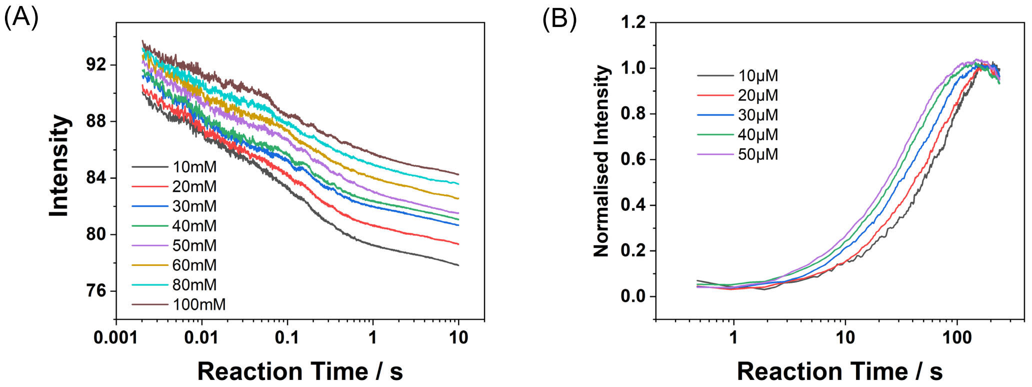

Figure S2. (A) Kinetic raw traces of Cu^{2+} (1 μM) binding to NBD labelled SUVs (100 μM total lipid) under different HEPES concentrations. (B) Reaction traces of SUV-Cu^{2+} conjugate (1 μM Cu^{2+} pre-mixed with 100 μM total lipid) with different concentrations of EDTA. The experiments were performed in HEPES and 100 mM NaCl buffer solution at 298 K (pH 7.5).

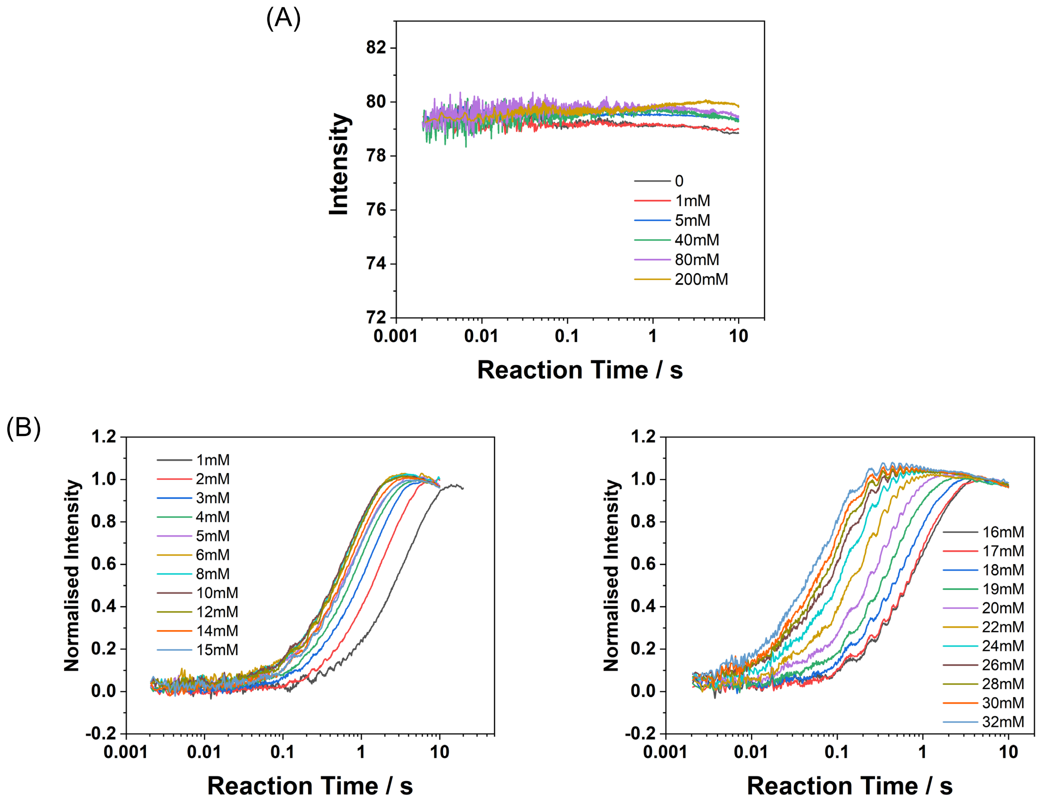

Figure S3. (A) Reduction traces of SUV-Cu^{2+} conjugate by various concentrations of ascorbate showing no reduction activity. (B) Reduction traces of SUV-Cu^{2+} conjugate by various concentrations of GSH. The experiments were performed in 50 mM HEPES and 100 mM NaCl buffer solution at 298 K (pH 7.5).

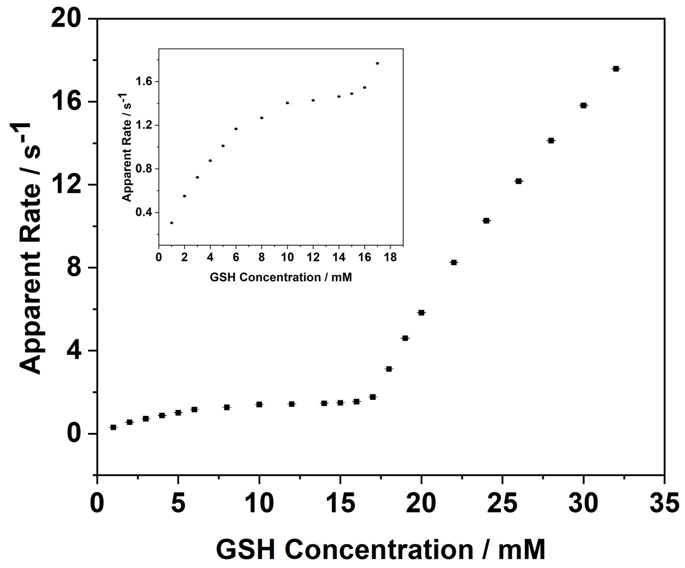

Figure S4. GSH concentration dependence of apparent reduction rates of WT-αSyn-Cu^{2+} complex (50 nM). The inset shows the expanded view of the rates under GSH concentration between 1 mM and 17 mM.

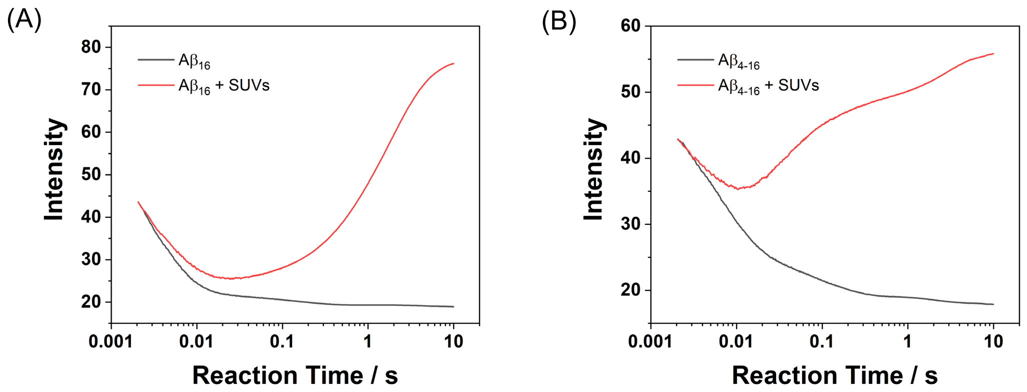

Figure S5. Competition of Cu^{2+} binding (1 µM Cu^{2+}) between synaptic-like SUVs (100 µM total lipid) and 25 nM (A) $Aβ_{16}$ as well as (B) $Aβ_{4-16}$. The experiments were performed in 50 mM HEPES and 100 mM NaCl buffer solution at 298 K (pH 7.5).

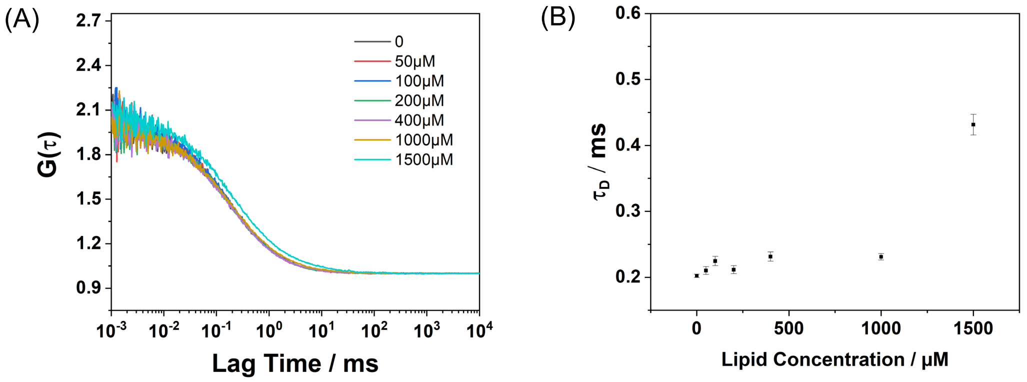

Figure S6. FCS measurements for labelled $Aβ_{40}$ in the presence of different concentrations of SUV lipid showing negligible interaction between synaptic-like SUVs and $Aβ_{40}$. (A) Normalised FCS curves. (B) Diffusion times obtained from the FCS curves. The experiments were performed in 50 mM HEPES and 100 mM NaCl buffer solution at 298 K (pH 7.5).

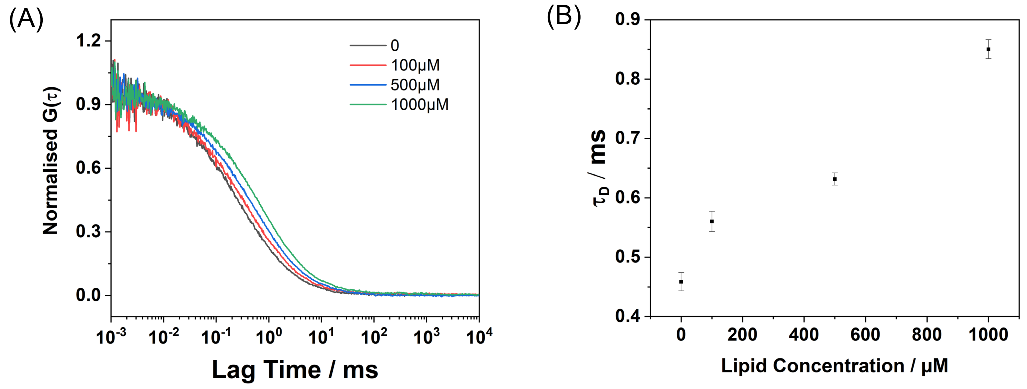

Figure S7. FCS measurements for labelled HSA in the presence of different concentrations of SUV lipid showing negligible HSA binding to synaptic-like SUVs. (A) Normalised FCS curves. (B) Diffusion times obtained from the FCS curves. The experiments were performed in 50 mM HEPES and 100 mM NaCl buffer solution at 298 K (pH 7.5).

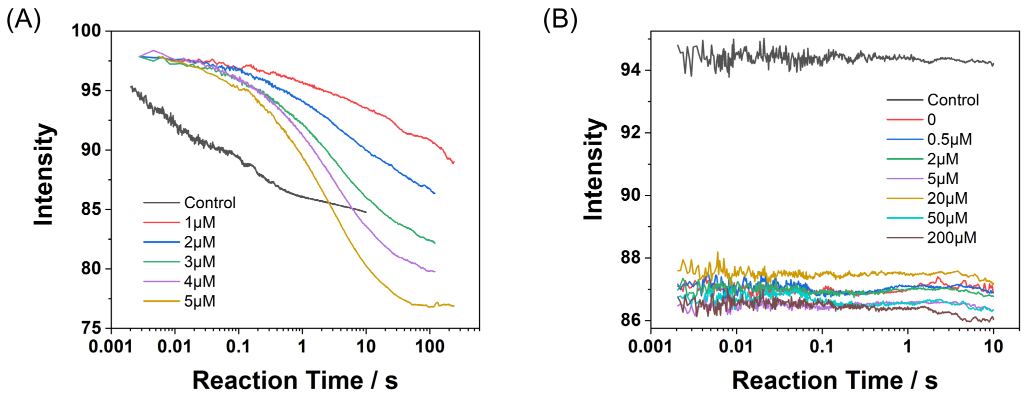

Figure S8. (A) Cu^{2+} competition between HSA (5 μM) and synaptic-like SUVs (100 μM total lipid) under various concentrations of Cu^{2+}. The control trace represents 1 μM Cu^{2+} binding to SUVs (100 μM total lipid) without HSA. (B) Cu^{2+} extraction from SUV-Cu^{2+} conjugate by different concentrations of HSA. The control trace represents the signal of labelled SUVs (100 μM total lipid) after mixing with 5 μM HSA. The experiments were performed in 50 mM HEPES and 100 mM NaCl buffer solution at 298 K (pH 7.5).

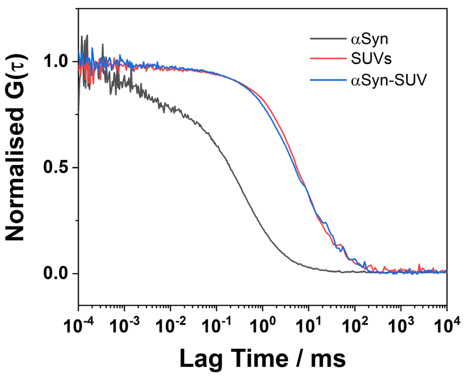

Figure S9. FCS traces showing that αSyn (fluorescently labelled, 10 nM) can bind to synaptic-like SUVs (1 mM total lipid). The experiments were performed in 50 mM HEPES and 100 mM NaCl buffer solution at 298 K (pH 7.5).

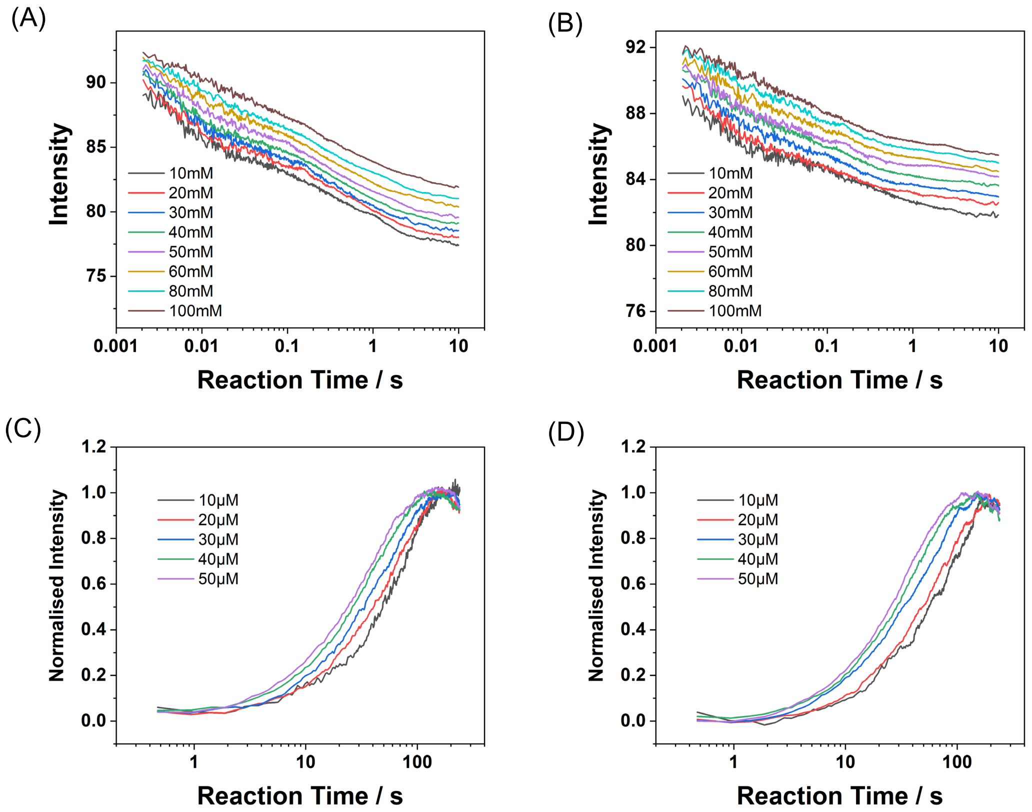

Figure S10. Kinetic raw traces of Cu^{2+} binding to (A) WT-αSyn-SUV and (B) NAc-αSyn-SUV conjugates under different HEPES concentrations, and reaction traces of (C) WT-αSyn-SUV-Cu^{2+} and (D) NAc-αSyn-SUV-Cu^{2+} conjugates (25 nM labelled αSyn pre-mixed with 100 μM total lipid, and then blended with 1 μM Cu^{2+}) with different concentrations of EDTA. The experiments were performed in HEPES and 100 mM NaCl buffer solution at 298 K (pH 7.5).

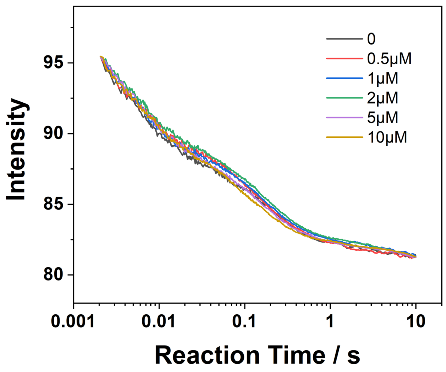

Figure S11. Cu^{2+} (1 μM) binding to NAc-αSyn-SUV conjugate under great excess of NAc-αSyn (various αSyn concentrations). The experiments were performed in 50 mM HEPES and 100 mM NaCl buffer solution at 298 K (pH 7.5).

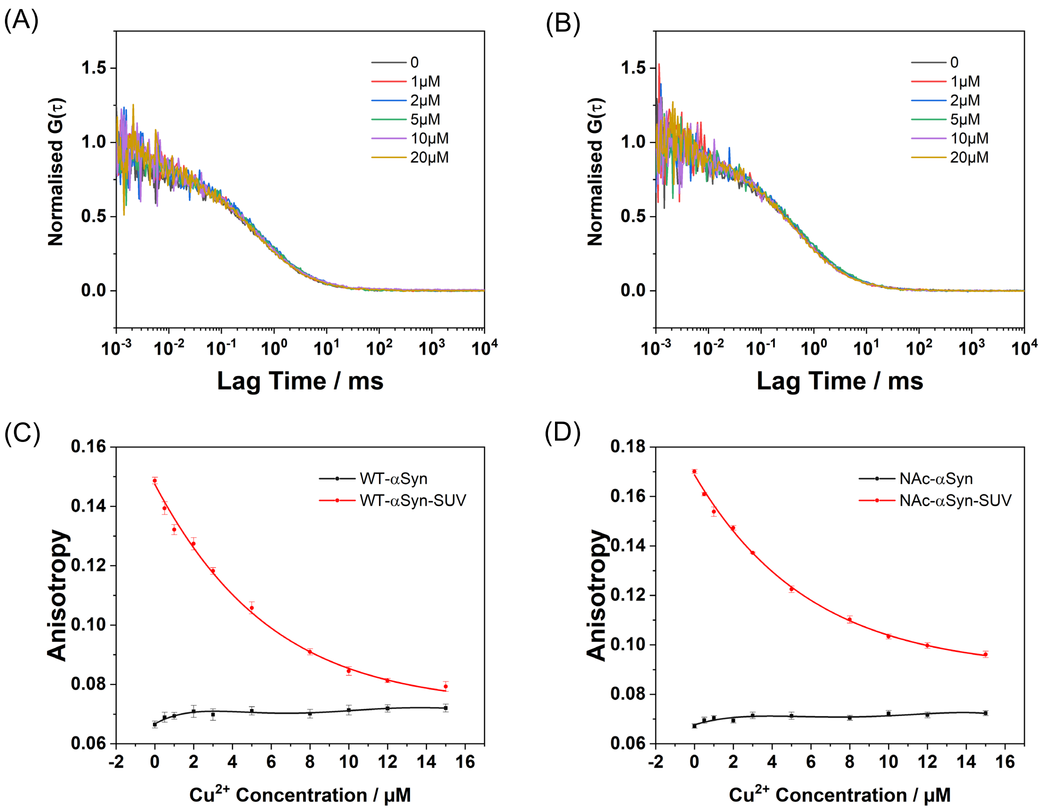

Figure S12. FCS measurements for various concentrations of Cu^{2+} binding to (A) WT-αSyn and (B) NAc-αSyn showing that Cu^{2+} caused fluorescence quenching has no impact on FCS signal recording. Fluorescence anisotropy titrations for various concentrations of Cu^{2+} binding to (C) WT-αSyn and (D) NAc-αSyn in the absence and presence of SUVs showing that Cu^{2+} can detach αSyn from αSyn-SUV conjugate. The experiments were performed in 50 mM HEPES and 100 mM NaCl buffer solution at 298 K (pH 7.5).

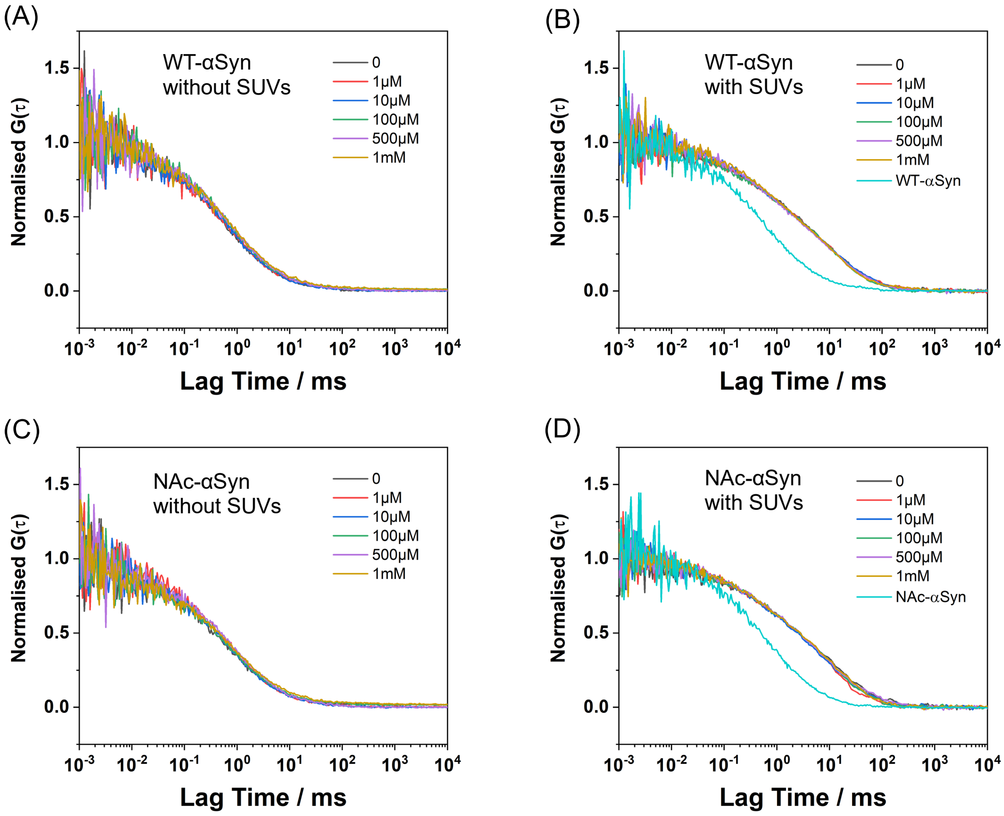

Figure S13. FCS measurements for the interactions of WT-αSyn ((A) & (B)) and NAc-αSyn ((C) & (D)) with various concentrations of Ca^{2+} in the absence and presence of SUVs showing that Ca^{2+} cannot detach αSyn from αSyn-SUV conjugate. The experiments were performed in 50 mM HEPES and 100 mM NaCl buffer solution at 298 K (pH 7.5).

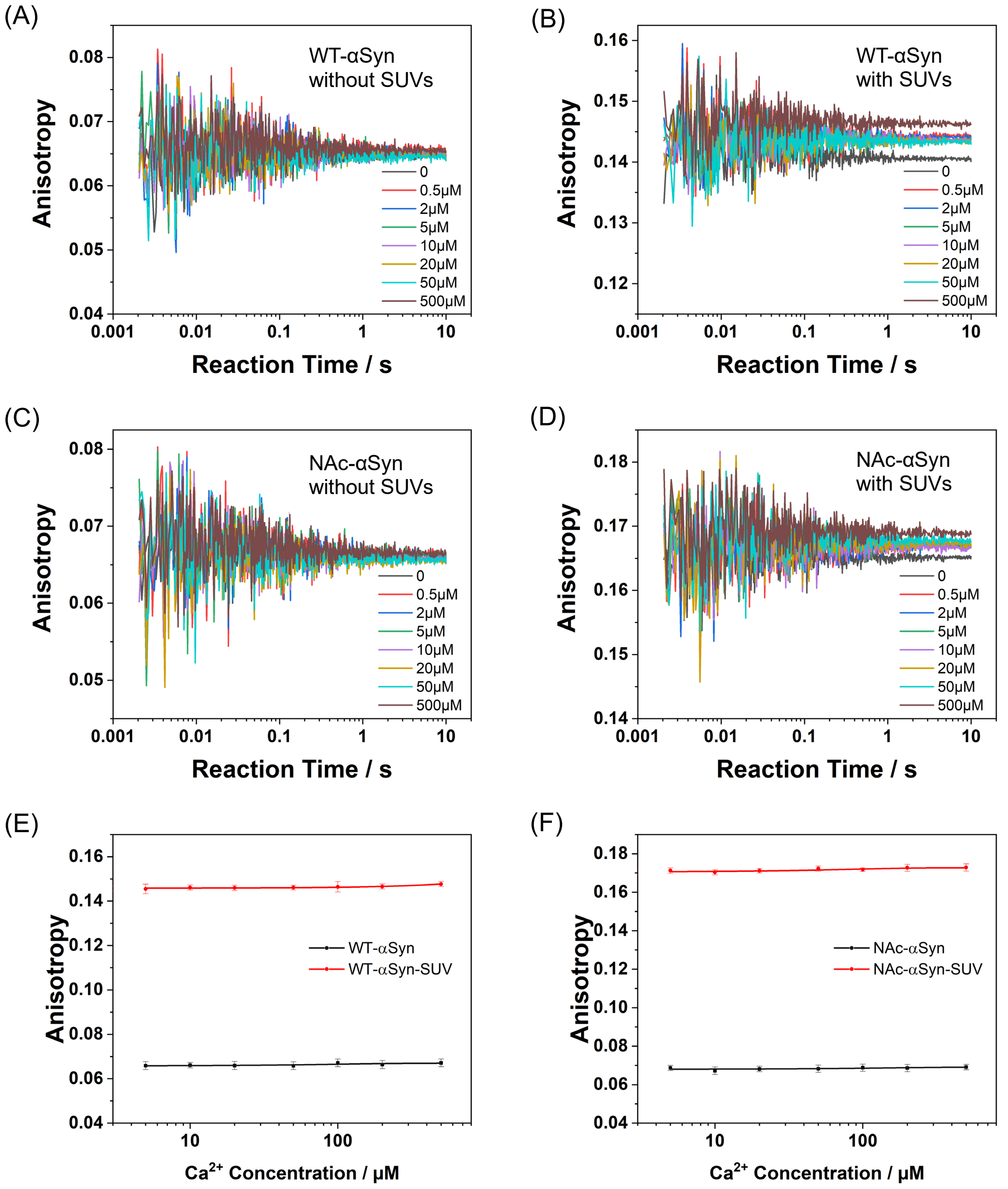

Figure S14. Kinetic fluorescence anisotropy assays ((A) to (D)) and fluorescence anisotropy titrations ((E) & (F)) for the interactions of WT-αSyn and NAc-αSyn with various concentrations of Ca^{2+} in the absence and presence of SUVs showing that Ca^{2+} cannot detach αSyn from αSyn-SUV conjugate. The experiments were performed in 50 mM HEPES and 100 mM NaCl buffer solution at 298 K (pH 7.5).

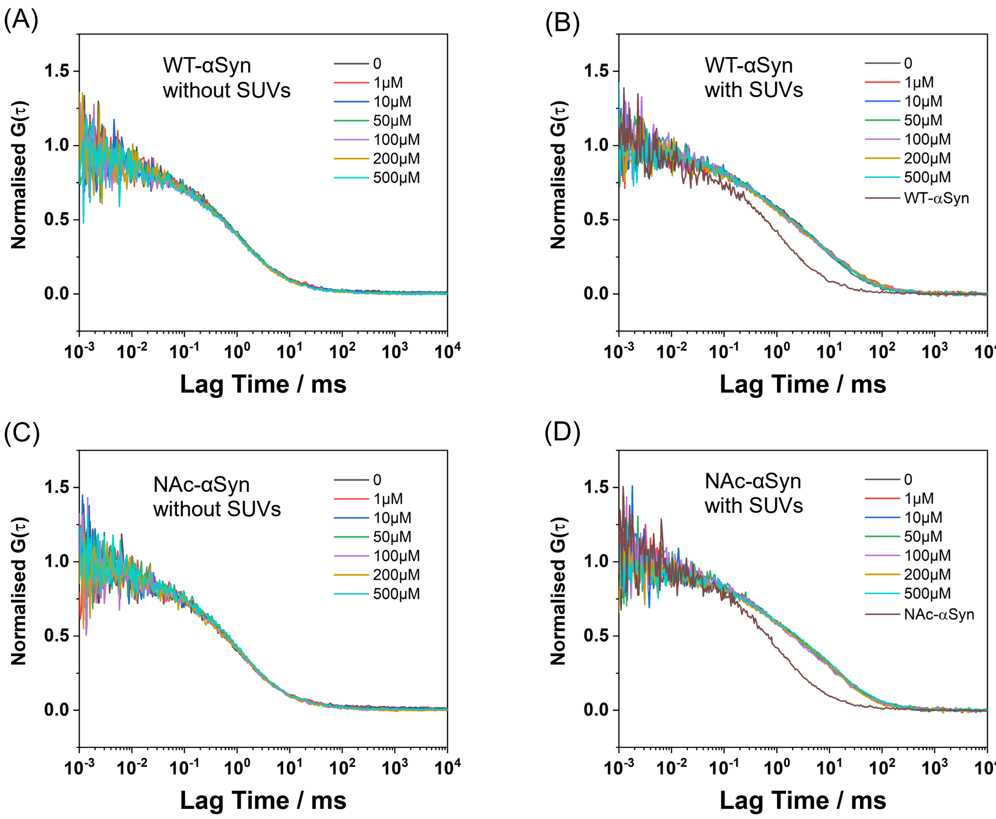

Figure S15. FCS measurements for the interactions of WT-αSyn ((A) & (B)) and NAc-αSyn ((C) & (D)) with various concentrations of Zn^{2+} in the absence and presence of SUVs showing that Zn^{2+} cannot detach αSyn from αSyn-SUV conjugate. The experiments were performed in 50 mM HEPES and 100 mM NaCl buffer solution at 298 K (pH 7.5).

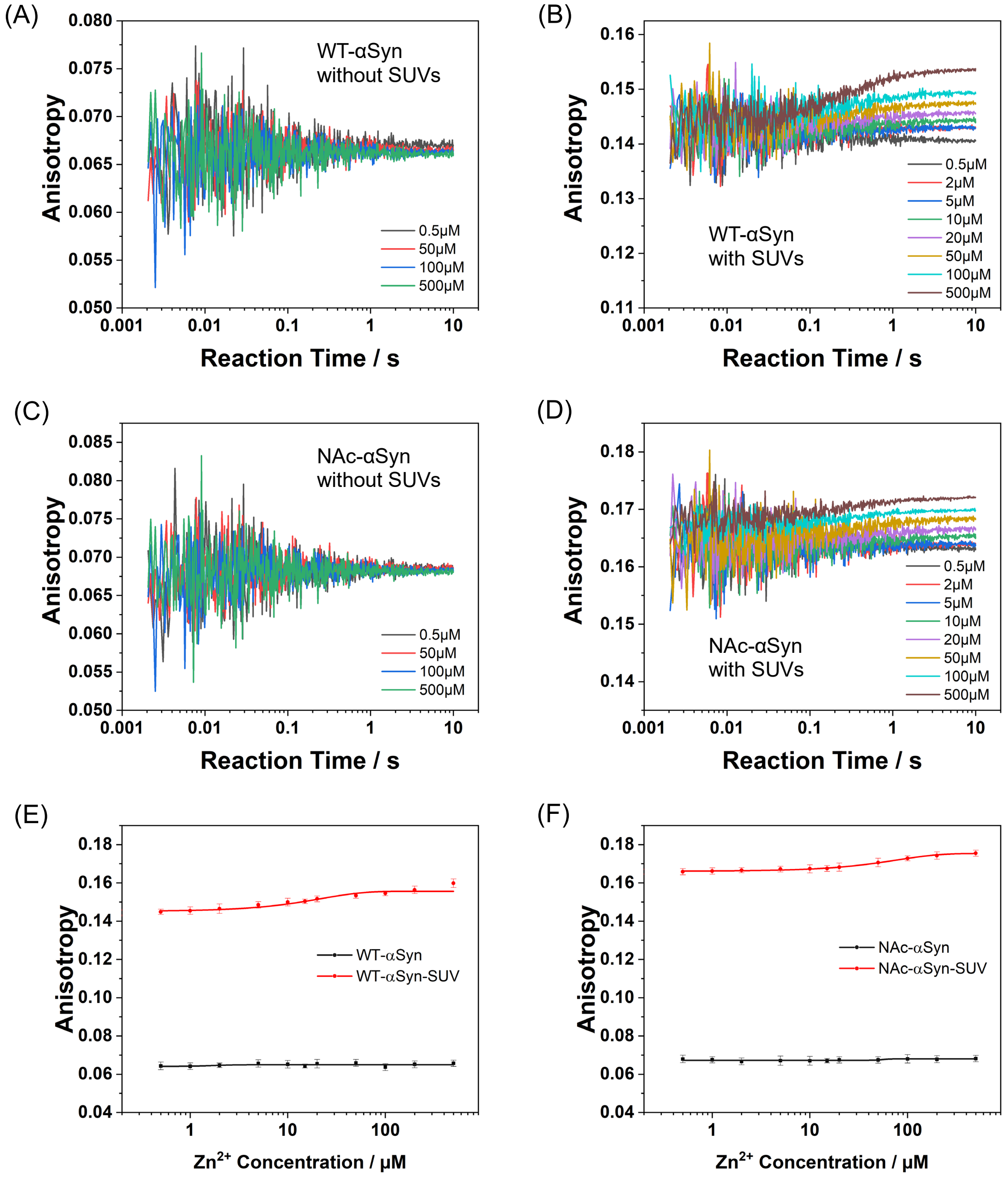

Figure S16. Kinetic fluorescence anisotropy assays ((A) to (D)) and fluorescence anisotropy titrations ((E) & (F)) for the interactions of WT-αSyn and NAc-αSyn with various concentrations of Zn^{2+} in the absence and presence of SUVs showing that Zn^{2+} cannot detach αSyn from αSyn-SUV conjugate. The experiments were performed in 50 mM HEPES and 100 mM NaCl buffer solution at 298 K (pH 7.5).

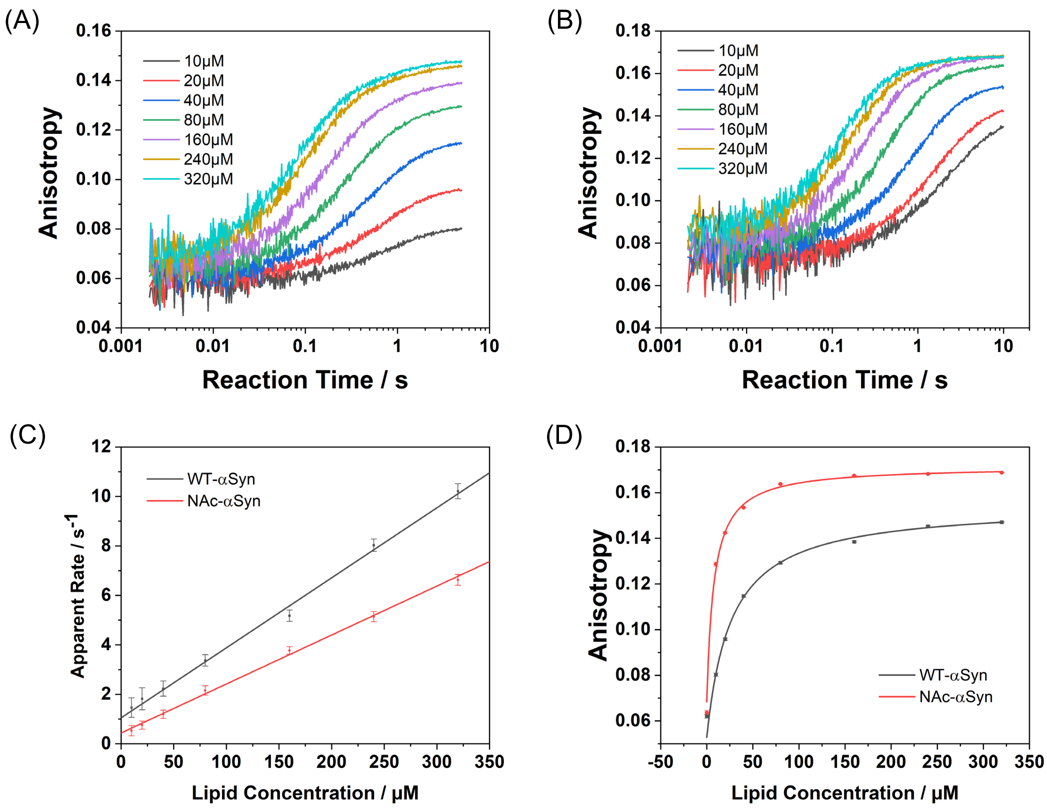

Figure S17. Kinetic fluorescence anisotropy curves of WT-αSyn (A) and NAc-αSyn (B) binding to various concentrations of SUV lipid. (C) Apparent binding rates of αSyn to SUVs derived from fluorescence anisotropy kinetic curves. (D) Final anisotropy from the fits of fluorescence anisotropy kinetic curves as a function of SUV lipid concentration. The experiments were performed in 50 mM HEPES and 100 mM NaCl buffer solution at 298 K (pH 7.5).
